# Modelling Resistance Management Benefits of Incomplete Cross‐Resistance Within a Fungicide Mode of Action Group

**DOI:** 10.1111/eva.70346

**Published:** 2026-11-05

**Authors:** Isabel Corkley, Neil D. Paveley, Bart A. Fraaije, Joseph Helps, Michael W. Shaw, Alice E. Milne, Nichola J. Hawkins, Frank van den Bosch

**Affiliations:** ^1^ Rothamsted Research Harpenden Hertfordshire UK; ^2^ ADAS Wolverhampton UK; ^3^ School of Agriculture, Policy and Development University of Reading Reading Berkshire UK; ^4^ BU Biointeractions and Plant Health, Wageningen Plant Research Wageningen University and Research Wageningen the Netherlands; ^5^ NIAB Cambridge UK; ^6^ Quantitative Biology & Epidemiology Group, Plant Pathology Department University of California, Davis Davis California USA

**Keywords:** CYP51, epidemiological model, fungicide resistance, haplotype, partial cross‐resistance

## Abstract

Mixing fungicides with different modes of action (MoA) is a well‐established resistance management tactic which relies on the general absence of cross‐resistance between MoAs. Mixtures of fungicides with the same MoA and complete cross‐resistance are not expected to contribute to resistance management. However, in some cases cross‐resistance is only partial within a MoA, for example azole fungicides in the demethylation inhibitor (DMI) group. We developed and used a mathematical population genetic model to explore possible resistance management benefits of mixtures of fungicides with the same MoA with incomplete cross‐resistance. An important example in agriculture is the deployment of DMIs against the wheat pathogen *Zymoseptoria tritici*. We modelled selection for known *Z. tritici* CYP51 haplotypes following application of solo azoles or two, three or four‐way mixtures of epoxiconazole, prothioconazole, prochloraz and tebuconazole. The resulting rate of resistance development was measured as the rate of change in the pathogen population growth rate, $∆\bar{r}$: this indicates how fast the level of disease severity will increase as resistance develops. We focused on within‐MoA programmes to investigate resistance management benefits of within‐MoA active substance diversity; in practice azoles should be used in combination with other MoA wherever possible. Our results show that mixtures of fungicides with incomplete cross‐resistance within a MoA can contribute to resistance management, achieving greater stability of disease control. Increasing the number of azole active substances available to mix in various combinations reduced $∆\bar{r}$ on average, but with diminishing returns. A generalised linear model fitted to the population genetic model results showed that $∆\bar{r}$ increased with the average level of disease control and with the maximum variance in haplotype sensitivity to an individual azole component. Azole combinations with low or negative cross‐resistance maximised resistance management benefits, but any degree of incomplete cross‐resistance was useful.

## 1 Introduction

Mixing fungicides with different modes of action (MoAs) is a proven resistance management tactic to slow the rate of evolution in crop pathogens (Taylor and Cunniffe 2023b; van den Bosch, Oliver, et al. 2014; van den Bosch, Paveley, et al. 2014). This tactic works because there is generally a lack of cross‐resistance between MoAs. Pathogen strains that are resistant to one MoA are controlled by the other MoA in the mixture, reducing the difference in population growth rates of resistant and sensitive strains. This reduces the selective advantage of fungicide resistance. Mixture of different MoAs can therefore reduce the rate at which resistance genes are selected for in the pathogen population.

The level of cross‐resistance in a pathogen population to two fungicides is measured by the correlation of the sensitivity of pathogen strains to each fungicide. Strong positive cross‐resistance implies that pathogen strains that are sensitive to one fungicide are also sensitive to the second fungicide, and that strains with the lowest level of sensitivity to one fungicide are also the least sensitive to the second fungicide. Mixtures of active substances within a MoA are not usually recommended for resistance management, as there is typically strong positive cross‐resistance of pathogen strain sensitivity between active substances in the same MoA. Cross‐resistance is complete if there is perfect positive correlation of the sensitivity of all pathogen strains to both active substances. Incomplete cross‐resistance occurs when there is either low or negative correlation between the sensitivity of pathogen strains to two fungicides. It has been hypothesised that mixtures of active substances within a MoA where cross‐resistance is incomplete could contribute to fungicide resistance management. However, there is limited evidence to demonstrate potential resistance management benefits of mixtures of active substances with incomplete cross‐resistance within a MoA, or to show how within‐MoA mixtures could best be deployed to maximise any such benefits. In this study, we use population genetic modelling to investigate whether use of within‐MoA mixtures with incomplete cross‐resistance could be a useful resistance management strategy, and to investigate how the number of active substances available within a MoA and the degree of cross‐resistance between active substances in a MoA affect the likely success or otherwise of this strategy.

A key example in agriculture of incomplete cross‐resistance within a MoA is the azoles in the demethylation inhibitor (DMI) group of fungicides (FRAC Code 3) (Dooley et al. 2016; EPPO Workshop 2010). All azoles share the same single target‐site: inhibition of the sterol 14a‐demethylase (CYP51) enzyme which catalyses the conversion of lanosterol into ergosterol; an essential component of fungal cell membranes (Rodrigues 2018). Inhibition of this metabolic pathway impacts on other cellular processes through production of toxic diol byproducts and activation of cell death pathways (Kelly et al. 1995; Schuster et al. 2024), resulting in reduced fungal population growth rates. Patterns of cross‐resistance between fungicides in the demethylation inhibitor (DMI) group include both cases described above—an absence of cross‐resistance and strong positive cross‐resistance—in addition to weak positive cross‐resistance and negative cross‐resistance (where a mutation that increases resistance to one azole increases sensitivity to another) (Dooley et al. 2016; Fraaije et al. 2007; Jørgensen et al. 2021; Leroux and Walker 2011). The primary cause of azole resistance in many pathogens is CYP51 target‐site alteration, although target‐site over‐expression, increased non‐target‐site efflux pump activity and suppression of other enzymes involved in ergosterol biosynthesis are also implicated (Cools and Fraaije 2013; Kelly et al. 1995; Leroux and Walker 2011). The effects of target‐site mutations vary between azole fungicides due to structural differences at a molecular level and variation in the binding activity of different azole ligands with regions involved in or bordering the CYP51 enzyme active site (Mullins et al. 2011; Xiao et al. 2004). For example, the amino acid substitution I381V decreases pathogen sensitivity to tebuconazole, but has little effect on sensitivity to epoxiconazole, and increases sensitivity to prochloraz (Fraaije et al. 2007; Mullins et al. 2011; Stammler et al. 2008). Interactions between multiple CYP51 mutations can also affect the sensitivity phenotype.

*Zymoseptoria tritici*, a polycyclic foliar fungal pathogen which causes the economically damaging disease septoria tritici blotch (STB) in wheat, has accumulated large numbers of target‐site alterations of the CYP51 protein. The *Z. tritici* population in Europe is composed of multiple strains, each with different combinations of these target‐site mutations and differing levels of sensitivity to each azole active substance. In field experimentation, Heick et al. (2017) found that increased diversity of azole active substances in application programmes reduced selection overall for CYP51 alterations, whereas Dooley et al. (2016) found that mixtures of epoxiconazole and metconazole did not reduce selection for azole‐resistant *Z. tritici* strains when compared to solo application (at the same total azole dose) of either fungicide. Differential effects of azoles on selection for strains with different CYP51 combinations have also been observed in *Phakopsora pachyrhizi* (the cause of Asian soybean rust) and *Cercospora beticola* (the cause of cercospora leaf spot of sugar beet) (Heick et al. 2017; Kayamori et al. 2021; Stammler et al. 2008; Stilgenbauer et al. 2023).

To model the resistance management benefit of a fungicide mixture, an estimate of the effect of the mixture on pathogen growth rates is needed, as the difference in the growth rates of resistant and sensitive strains determines the strength of selection for resistance (van den Bosch, Oliver, et al. 2014). Existing models of the joint action in mixture of two or more fungicides on pathogen growth rates typically use various forms of either an additive dose model (ADM) or a multiplicative survival model (MSM). These describe alternative methods of combining the dose–response curves of the fungicides (when applied solo) to estimate the effect of the mixture on pathogen survivorship, growth rates or life cycle parameters. In ADMs, the effects of the doses of each fungicide in the mixture are added together. Dose rates of each fungicide calculated from the solo dose–response curves as giving equivalent levels of control could be substituted for each other with no change in the overall level of control of the mixture. In the MSM, the fractional survivorship following application of each fungicide is multiplied together to give the combined survivorship that results from the mixture application. The ADM is typically applied to mixtures within a MoA, and the MSM to mixtures of different MoA (Paveley et al. 2003). If cross‐resistance within a MoA is close to complete, then the ADM is appropriate: that is, mixtures within the MoA are the equivalent of mixing the same active substance with itself, with no resistance management benefit (Oliver 2016). If cross‐resistance is weak, then the effects of mixture within a MoA would be closer to those described by the MSM. The joint action of fungicides within a MoA with incomplete cross‐resistance, as observed for azole fungicides, cannot be adequately described by either the ADM or MSM. We describe here a method to address this limitation.

The datasets generated through sampling and study of *Z. tritici* isolates make this pathogen a tractable case study for parameterisation of a model of the effects of within‐MoA mixtures. *Zymoseptoria tritici* has both sexual and asexual reproductive cycles, but the majority of infection during the growing season is driven by asexual haploid pycnidiospores, spread between leaves by contact and rain splash (Shaw and Royle 1993). We can therefore consider the effect of the haploid CYP51 genotype (‘haplotype’) on the growth rate of strains in the presence of fungicides—that is, the effect of single chromosomes rather than pairs of chromosomes. We refer to CYP51 variants with particular combinations of alterations as ‘haplotypes’. A few key CYP51 haplotypes typically comprise a high proportion of the *Z. tritici* population in any season (Glaab et al. 2024; Kildea et al. 2023). The laboratory sensitivities of these key haplotypes to contrasting azoles have been determined (Cools et al. 2011; Huf et al. 2018; Kildea et al. 2023; Kirikyali et al. 2017), and recent analysis of long‐term datasets has related laboratory to field sensitivities (Blake et al. 2018), enabling parameterisation of the field sensitivities of haplotypes for a population genetic approach.

We introduce a population genetic model that tracks CYP51 haplotype frequencies in the pathogen population under a range of azole fungicide treatment programmes, with a sub‐model introduced to account for the joint action of multiple azoles. Our approach explicitly models the growth of individual haplotypes and fungicide dose decay, enabling modelling of a wide range of fungicide programmes. We introduce the use of the rate of change in the average growth rate of the entire pathogen population, $∆\bar{r}$, to assess selection for fungicide resistance in pathogen populations with multiple genotypes, each with a different level of resistance. $∆\bar{r}$ has practical relevance to farmers since it describes how fast the epidemic growth rate encountered in the field changes due to the development of resistance. The model and its application to simulate azole mixture, alternation and mosaic programmes were previously described in Corkley et al. (2023) and Corkley (2025). Here we describe the model derivation and parameterisation for *Z. tritici* CYP51 haplotypes in full, and use the model to investigate resistance management benefits of mixtures within a MoA with incomplete cross‐resistance to answer the following questions:

- Are there resistance management benefits of mixture within the same mode of action?
- How do resistance management benefits vary with the number of active substances available within a mode of action?
- How do resistance management benefits vary with the degree of cross‐resistance between active substances?

## 2 Materials and Methods

### 2.1 Measuring Resistance Management Benefits

#### 2.1.1 Selection for Fungicide Resistance: Rate of Change in Epidemic Growth Rate

Each CYP51 haplotype has its own population growth rate, $r_{i}$, under an azole fungicide treatment regime (Figure 1a). Haplotypes with lower sensitivity to a fungicide will have the highest growth rates in the presence of that fungicide. These differences in population growth rate cause the frequencies of the haplotypes in the pathogen population to change. The larger the difference between the sub‐population growth rate of a haplotype, $r_{i}$, and the mean total pathogen population growth rate, $\bar{r}$, the greater the rate of selection for or against that haplotype.

**FIGURE 1:**
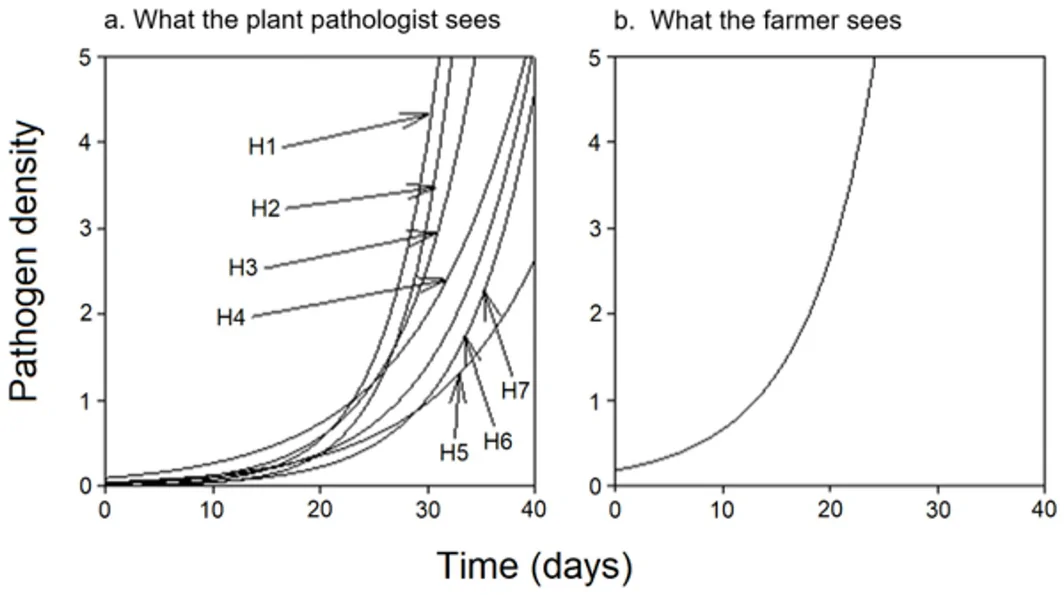
Epidemic growth rates: What a plant pathologist sees versus what a farmer sees. (a) Sub‐populations of seven pathogen haplotypes (H1–H7), each with a different initial density and a different sub‐population growth rate, $r_{i}$, are shown. (b) The epidemic the farmer observes in the field is the sum of the densities of all haplotypes, with average growth rate $\bar{r}$.

The population growth of each haplotype can be calculated and the frequency of it in the total pathogen population tracked through time. Such ensembles of haplotypes and their population dynamics are of interest to plant pathologists and theoretical biologists. However, ‘resistance frequency’ cannot be used as a meaningful single measure in the context of a set of strains, each with a different level of resistance, so is not suitable to communicate the rate of resistance development to farmers in this case. EC_50_ (and the corresponding field‐ED_50_) is a useful measure for plant pathologists to compare the resistance‐related component of the relative fitness of haplotypes, and could be summarised at a population level through frequency‐weighting, but the practical relevance for farmers is still limited: the size of the EC_50_ shift is not a consistent measure of the impact of resistance development, as a shift from low to moderate EC_50_ has a greater effect on disease control than a shift from high to very high EC_50_.

The average growth rate of the entire pathogen population, $\bar{r}$, in fungicide‐treated crops is a single measure covering all pathogen strains that summarises the level of resistance of the population and has practical relevance to farmers, since the epidemic growth rate determines the level of damage to the crop (Figure 1b). Since fungicide applications select for the strains that are less sensitive to fungicide, over time they select for an increase in the average epidemic growth rate, $\bar{r}$. Resistance management aims to minimise this increase. We therefore use the rate of change in $\bar{r}$ from 1 year to the next, $∆\bar{r}$, as a single measure of selection for fungicide resistance which has practical relevance to farmers, as it measures how fast the disease severity seen in the field will change due to the development of resistance. Denoting the growth rate of the pathogen population in year *n* as ${\bar{r}}_{n}$, the rate of resistance development is measured as:

(1) $$∆\bar{r}=\frac{\bar{r_{n+1}}-\bar{r_{n}}}{\bar{r_{n}}}$$

A small value of $∆\bar{r}$ indicates that the epidemic growth rate is only changing slowly, meaning that the average level of disease control remains relatively stable over time. A high value of $∆\bar{r}$ indicates that the epidemic growth rate is increasing rapidly due to selection for more resistant pathogen strains. A large shift in the frequency‐weighted EC_50_/field‐ED_50_ of the entire pathogen population could result in a high or moderately high value of $∆\bar{r}$, depending on the impact of the shift on disease control (Table S.7, Supplementary Material S5).

#### 2.1.2 Threshold for Effective Disease Control

An effective fungicide programme is one that prevents economically damaging levels of disease. We follow previous studies (Gladders et al. 2001; te Beest et al. 2009) in defining a damaging epidemic as one where Z. *tritici* severity on the upper three leaves of the wheat crop canopy exceeds 5% during grain filling (growth stage 75 (GS75); Zadoks et al. (1974)). We therefore use 5% severity at GS75 as a threshold value, $S_{\mathrm{Max}}$, to measure whether a fungicide programme provides effective disease control.

### 2.2 The Population Genetic Model

#### 2.2.1 CYP51 and the Azole Dose–Response Curve

The CYP51 enzyme takes part in the conversion of lanosterol into ergosterol by removal of the 14α‐methyl group from sterol substrates (Hargrove et al. 2018). Ergosterol is an essential building block of cell membranes and acts to maintain membrane integrity (Rodrigues 2018). Azole fungicides affect pathogen fitness by targeting the CYP51 enzyme. We assume that azole fungicides act as competitive CYP51 inhibitors which bind to the enzyme and occupy the P450 active site, preventing substrate binding. The resulting reduction in ergosterol production and related impacts on cell processes causes a reduction in the fungal population growth rate.

Lanosterol binds to the enzyme at rate $k_{1}$, and either unbinds from the enzyme at rate $k_{-1}$, or is converted to ergosterol at rate $k_{2}$. The binding of an azole molecule to the enzyme at rate $k_{3}$ is reversible, so the azole molecule can unbind at rate $k_{-3}$ (Supplementary Material S1, Figure S.1). Given the timescale difference between these molecular level processes and the population dynamic processes relevant to the pathogen dose–response curve, we can assume that this process is in quasi‐steady‐state (Michaelis and Menten 1913; Mikaberidze et al. 2017) and can calculate the rate of ergosterol production, *V*, from Hill's equation (Hill 1913) as used in enzyme kinetics studies:

(2) $$\begin{array}{c} V=\frac{A}{1+\mathrm{BD}}\; \end{array}$$

where $A=\frac{k_{1}LE_{0}}{k_{-1}+k_{2}+k_{1}L}$ and $B=\frac{k_{3}}{k_{-3}}\frac{k_{-1}+k_{2}}{k_{-1}+k_{2}+k_{1}L}$, where L is the concentration of lanosterol, $E_{0}$ is the total concentration of CYP51 enzyme (free and bound), B is the dose–response parameter for the azole fungicide and D is the fungicide dose.

Assuming competitive binding of azole molecules, meaning that when an azole molecule is bound to the CYP51 enzyme, other azole molecules cannot bind to that CYP51 molecule, we generalise Equation (2) for the rate of ergosterol production, *V*, to mixtures of azoles:

(3) $$\begin{array}{c} V=\frac{A}{1+B_{1}D_{1}+B_{2}D_{2}} \end{array}$$

where $B_{1}$ and $B_{2}$ are the dose–response parameters for azole 1 and azole 2 respectively and $D_{1}$ and $D_{2}$ are the doses of azole 1 and azole 2, respectively. An illustration of the dose–response curve of the ergosterol production rate, V, to a mixture of two azoles for varying values of $B_{1}$ and $B_{2}=1$ is shown in Figure S.2 in Supplementary Material S1. Note that although we show here the derivation of the dose–response curve for competitive inhibition, the form of the dose–response curve is the same for non‐competitive inhibition (see Section S.1.1 in Supplementary Material S1).

Equation (3) can be generalised to include more than two azoles and multiple pathogen haplotypes. For the purpose of modelling the effects of azole fungicides, we assume that the only process varying between haplotypes is the ergosterol production rate and that CYP51 inhibition is the sole mode of action of azole fungicides against the pathogen. We can therefore describe the population growth rate of pathogen haplotype i, $r_{i}$, as affected by azole fungicides:

(4) $$\begin{array}{c} r_{i}=\frac{r_{0}}{1+\sum_{j=1}^{\text{jmax}}B_{\mathrm{ij}}D_{j}} \end{array}$$

where $r_{0}$ is the growth rate of the pathogen when no fungicide is applied, the subscript j denotes each azole fungicide, the number of azoles used is jmax, $D_{j}$ is the dose of azole j and $B_{\mathrm{ij}}$ describes the effect of azole j on the growth rate of haplotype i. For haplotypes with one or more mutations in the gene coding for the CYP51 enzyme, binding of the azole to CYP51 will be more difficult, and/or make unbinding easier, equating to a smaller value of $B_{\mathrm{ij}}$ (i.e., less inhibition of the enzyme function). We assume that that there is no fitness cost due to the development of resistance, so $r_{0}$ is the same for all haplotypes: that is, when no fungicide is present, all pathogen haplotypes have the same population growth rate. Equation (4) thus defines the dose–response curve for the growth rate of all pathogen haplotypes and all azole treatments.

#### 2.2.2 The Growth Rate of the Sub‐Population of a Haplotype Following Azole Application

The model defines the fungicide dose as a function of time: $D_{j}\left(t\right)$ is the dose of azole *j* at time *t*, expressed as a proportion of ${D_{j}}_{\max}$, the maximum permitted individual dose (as defined on the fungicide product label). We assume that the concentration of the fungicide immediately following application is the applied dose, or the applied dose plus any residual concentration from the previous application. After application we assume that the fungicide decays exponentially due to processes such as plant metabolism and UV degradation (Fantke and Juraske 2013; Hobbelen, Paveley, Fraaije, et al. 2011):

(5) $$\begin{array}{c} D_{j}\left(t\right)=\left\{\begin{array}{c} {D_{j}}_{0}e^{-\alpha \left(t-t^{*}\right)},\;t\geq t^{*}\\ \;0,\;t<t^{*} \end{array}\;\right. \end{array}$$

where $t^{*}$ is the time of the first application of azole *j*, $D_{j0}$ is the application dose of azole *j*, and α is the decay rate of the fungicide. If an additional application of fungicide *j* is made at $t^{**}$, the remaining dose from the application at $t^{*}$ is added to the dose from $t^{**}$ to calculate the total remaining dose.

In field experiments, fungicide efficacy is usually assessed through the effect of fungicide dose on the disease severity, after an interval of time following fungicide application. The fungicide dose determines the pathogen sub‐population growth rate, $r_{i}$, which in turn determines the population size/disease severity (Figure 1). Assuming that the pathogen population grows exponentially in time (i.e., that density dependence does not limit growth during the period relevant for fungicide activity), we can calculate disease severity at any point in time, *t*.

The dynamics of disease severity caused by haplotype i at time t, $S_{i}\left(t\right)$, as affected by the dose of a single azole, $D_{j}\left(t\right)$, are given by:

(6) $$\begin{array}{c} \frac{d\;S_{i}\left(t\right)}{d\;t}=r_{i}\left(D_{j}\left(t\right)\right)S_{i}\left(t\right) \end{array}$$

where $r_{i}\left(D_{j}\left(t\right)\right)$ is the growth rate of haplotype i, from Equation (4).

This model can be solved (see Section S.1.2 in Supplementary Material S1) to calculate the severity at the end of the growing season, t=T.

For one fungicide application at dose $D_{j0}$ at time $t^{*}$, the disease severity caused by haplotype i at the end of the growing season, $S_{i}\left(T\right)$, is given by:

(7) $$\begin{array}{c} S_{i}\left(T\right)=S_{i}\left(0\right)e^{r_{0}t^{*}}{\left(\frac{B_{\mathrm{ij}}D_{j0}+e^{\alpha \left(T-t^{*}\right)}}{B_{\mathrm{ij}}D_{j0}+1}\right)}^{\frac{r_{0}}{\alpha }} \end{array}$$

where $S_{i}\left(0\right)$ is the severity of haplotype i at the start of the simulation. For two applications at dose $D_{j0}$, one at $t^{*}$, the second at $t^{**}$:

(8) $$\begin{array}{c} S_{i}\left(T\right)=S_{i}\left(0\right)e^{r_{0}t^{*}}{\left(\frac{B_{\mathrm{ij}}D_{j0}+e^{\alpha \left(t^{**}-t^{*}\right)}}{B_{\mathrm{ij}}D_{j0}+1}\right)}^{\frac{r_{0}}{\alpha }}{\left(\frac{B_{\mathrm{ij}}D_{j0}\left(1+e^{-\alpha \left(t^{**}-t^{*}\right)}\right)+e^{\alpha \left(T-t^{**}\right)}}{B_{\mathrm{ij}}D_{j0}\left(1+e^{-\alpha \left(t^{**}-t^{*}\right)}\right)+1}\right)}^{\frac{r_{0}}{\alpha }} \end{array}$$

These equations can be generalised to include more than one azole. If the decay rates $\alpha_{j}$ of the azoles are unequal, it is not straightforward to solve the model Equation (6), and recourse to numerical solutions is required. In the case that the fungicide decay rates are equal, $\alpha_{1}=\alpha_{2}$, the model is solved with a generalisation of Equations (7) and (8) using the term $\sum_{j=1}^{\text{jmax}}B_{\mathrm{ij}}D_{j}$ as in Equation (4). For two applications of an azole mixture, one at $t^{*}$, the second at $t^{**}$:

(9) $$\begin{array}{c} S_{i}\left(T\right)=S_{i}\left(0\right)e^{r_{0}t^{*}}{\left(\frac{\sum_{j=1}^{\text{jmax}}B_{\mathrm{ij}}D_{j}+e^{\alpha \left(t^{**}-t^{*}\right)}}{\sum_{j=1}^{\text{jmax}}B_{\mathrm{ij}}D_{j}+1}\right)}^{\frac{r_{0}}{\alpha }}{\left(\frac{\sum_{j=1}^{\text{jmax}}B_{\mathrm{ij}}D_{j}\left(1+e^{-\alpha \left(t^{**}-t^{*}\right)}\right)+e^{\alpha \left(T-t^{**}\right)}}{\sum_{j=1}^{\text{jmax}}B_{\mathrm{ij}}D_{j}\left(1+e^{-\alpha \left(t^{**}-t^{*}\right)}\right)+1}\right)}^{\frac{r_{0}}{\alpha }} \end{array}$$

The overall disease severity at time T, $S\left(T\right)$, our measure of disease control for comparison to the threshold value for effective disease control (see Section 2.1.2) is calculated as:

(10) $$\begin{array}{c} S\left(T\right)=\sum_{i=1}^{M}S_{i}\left(T\right) \end{array}$$

where *M* is the total number of haplotypes in the population.

#### 2.2.3 Growth Rate of the Overall Pathogen Population

The total pathogen population growth rate, $\bar{r}$, is calculated as the average growth rate over all the haplotypes in the population:

(11) $$\begin{array}{c} \bar{r}=\frac{\ln\left(\theta_{1}e^{r_{1}T}+\theta_{2}e^{r_{2}T}+\ldots +\theta_{M}e^{r_{M}T}\right)}{T} \end{array}$$

where $\theta_{i}$ is the frequency of haplotype i, $r_{i}$ is the sub‐population growth rate of haplotype i, and T is the length of time simulated (see Section 2.3.1). The sub‐population growth rate of a haplotype is interpreted as the mean growth rate over the cropping season, $\bar{r_{i}}$. As the sub‐population growth rate of a haplotype varies during the growing season, due to the application and subsequent decay of the fungicide, we calculate the mean haplotype growth rate using the expressions:

(12) $$S_{i}\left(T\right)=S_{i}\left(0\right)e^{\bar{r_{i}}T}\;\text{and}\;S_{i}\left(T\right)=S_{i}\left(0\right)G_{i}$$

where the term $G_{i}$ can be derived from Equation (9), and the severity of haplotype i at the start of the simulation, $S_{i}\left(0\right)$ is calculated as $S_{i}\left(0\right)=I_{0}{\theta_{i}}_{\text{start}}$, where $I_{0}$ is the initial severity of the overall pathogen population at t=0 and ${\theta_{i}}_{\text{start}}$ is the proportion of haplotype i in the initial infection of the crop. It follows that:

(13) $$\begin{array}{c} \bar{r_{i}}=\frac{1}{T}\ln\left(\frac{S_{i}\left(T\right)}{S_{i}\left(0\right)}\right)=\frac{1}{T}\ln\left(G_{i}\right) \end{array}$$

The mean total population growth rate/fitness in the first growing season is given by:

(14) $$\begin{array}{c} \bar{r_{y1}}=\frac{1}{T}\ln\;\left(\sum_{i=1}^{M}{\theta_{i}}_{\text{start}}e^{\bar{r_{i}}T}\right) \end{array}$$

At the end of the first growing season, the haplotype frequencies are:

(15) $$\begin{array}{c} {\theta_{i}}_{\mathrm{end}}=\frac{{\theta_{i}}_{\text{start}}G_{i}}{\sum_{i=1}^{M}{\theta_{i}}_{\text{start}}G_{i}} \end{array}$$

The mean population growth rate in the second year is therefore:

(16) $$\begin{array}{c} \bar{r_{y2}}=\frac{1}{T}\ln\;\left(\sum_{i=1}^{M}{\theta_{i}}_{\mathrm{end}}e^{\bar{r_{i}}T}\right) \end{array}$$

Our measure of selection for fungicide resistance, the rate of increase in total population growth rate, $∆\bar{r}$, can therefore be calculated by substituting Equations (14) and (16) into Equation (1).

We note that $\bar{r}$ could alternatively be calculated as the instantaneous average growth rate, as typically used in population genetics, with minor differences in calculated values of $\bar{r}$ and $∆\bar{r}$ compared to Equation (11) (see Section S.1.3, Supplementary Material S1). We chose to use the mean growth rate over the cropping season for consistency with ‘what the farmer sees’ in the field (Figure 1).

### 2.3 Model Parameterisation

The model was implemented in R version 4.2.0 (R Core Team 2022) and parameterised for *Zymoseptoria tritici* on wheat. We used a literature search to estimate values of $r_{0}$, T, $I_{0}$ (Section 2.3.1) and α (Section 2.3.2). We used a dataset of CYP51 haplotype frequencies over time and the sensitivity of each haplotype to four azoles (triazoles epoxiconazole, prothioconazole and tebuconazole and the imidazole prochloraz) (Tables S.2–S.5 in Supplementary Material S3) to estimate values of ${\theta_{i}}_{\text{start}}$ and the dose–response parameters for each haplotype‐azole combination, $B_{\mathrm{ij}}$.

#### 2.3.1 Parameterisation of $r_{0}$, T and $I_{0}$

##### 2.3.1.1 Pathogen Population Growth Rate When No Fungicide Is Applied, *r*_0_

Epidemic growth rates in the absence of fungicide vary from year to year, depending on weather conditions amongst other factors (Gladders et al. 2001). We used an estimate of the average growth rate, $r_{0}$ = 0.1173 $t^{-1}$, that was calculated from a dataset of disease severity on untreated plots from multiple sites and years (Hobbelen, Paveley, and van den Bosch 2011; te Beest et al. 2009).

##### 2.3.1.2 Timing of Fungicide Applications and End of the Growing Season, T

We set the start of the simulation, t = 0, to correspond to GS32, when the upper three leaves of the wheat crop canopy start to emerge. We estimate T = 53 days, approximately corresponding to the timing of GS75 relative to GS32 (Corkley et al. 2025). We assume that the haplotype composition at GS75 will be representative of the haplotype frequencies at the start of the following growing season, as the wheat leaf canopy rapidly senesces after GS75, limiting epidemic growth rate due to density dependence and a lack of green leaf area.

##### 2.3.1.3 Initial Severity of the Overall Pathogen Population, $I_{0}$

The mean untreated disease severity at GS75 was estimated as 25.0% by van den Bosch et al. (2020), from a dataset of UK field trials covering 21 years and 80 site‐years. We used this estimate in combination with the values of $r_{0}$ and T to estimate a value for the initial severity of the overall pathogen population, $I_{0}$.

#### 2.3.2 Fungicide Decay Rate, α

The fungicide decay rate, α, is calculated from the foliar dissipation half‐life (‘DT50’) of the fungicide, which we estimated from information available in public domain sources, from a range of crops for tebuconazole, epoxiconazole, prochloraz and the active metabolite of prothioconazole, prothioconazole‐desthio (Table S.1, Supplementary Material S2). There is large variability in the DT50 values estimated in experimental studies for each azole. There were no significant differences in the mean DT50 values of the azoles considered in this study, which ranged from 7.09 to 7.93 days, with an overall average of 7.5 days. We therefore used an estimate of 7.5 days for all azoles in this study. The decay rate, α, is then calculated as:

(17) $$\begin{array}{c} \alpha =\frac{-\ln\left(0.5\right)}{\mathrm{DT}50}=0.0924\;t^{-1} \end{array}$$

#### 2.3.3 Initial Haplotype Frequencies, ${\theta_{i}}_{\text{start}}$

As shown by Equations ((14), (15), (16))–((14), (15), (16)), we can calculate the selection rate, $∆\bar{r}$, when the initial haplotype composition of the pathogen population is known. The initial frequency, ${\theta_{i}}_{\text{start}}$, of each *Z. tritici* haplotype, i, was estimated from a dataset of isolates sampled in fields around Rothamsted Research, UK in each of the years 2003 and 2010–2019 (Figure S.3 in Supplementary Material S2; Tables S.2–S.4 in Supplementary Material S3). We ran simulations using each year's estimated relative haplotype frequencies for all haplotypes for which in vitro EC_50_ dose–response measurements were available, to represent realistic starting haplotype compositions.

#### 2.3.4 Fungicide Dose–Response of Each Haplotype, $B_{\mathrm{ij}}$

We used in vitro EC_50_ dose–response measurements (Table S.4 in Supplementary Material S3; Figure S.4 in Supplementary Material S2) to parameterise the fungicide dose–response parameter, $B_{\mathrm{ij}}$, for each of 116 haplotype‐azole combinations (29 haplotypes and 4 azoles). To calculate $B_{\mathrm{ij}}$, a measure of the field dose–response is needed. Blake et al. (2018) developed log–log relationships between lab‐EC_50_ and field‐ED_50_ values for epoxiconazole and for prothioconazole‐desthio (Section S.2.3, Supplementary Material S2). The ED_50_ measure of field dose–response represents the applied fungicide dose, $D_{j0}$, that would, on average, reduce the disease severity by 50% relative to an untreated ($D_{j0}$ = 0) epidemic. $B_{\mathrm{ij}}$ can be calculated from these field‐ED_50_ values in combination with Equation (7). Since field‐ED_50_ is the dose, ${D_{\mathrm{ij}}}_{50}$, at which $S_{i}\left(T\right)=0.5S_{i0}e^{r_{0}T}$, it follows that:

(18) $$\begin{array}{c} B_{\mathrm{ij}}=\frac{a-e^{\mathrm{\alpha T}}}{{D_{\mathrm{ij}}}_{50}\left(1-a\right)} \end{array}$$

where $a={\left(0.5e^{r_{0}T}\right)}^{\frac{\alpha }{r_{0}}}$.

We used the relationships developed by Blake et al. (2018) to convert lab‐EC_50_ values into ${D_{\mathrm{ij}}}_{50}$ values for each haplotype. The relationship developed for epoxiconazole was also used for tebuconazole and prochloraz (see Section S.2.3 in Supplementary Material S2). $B_{\mathrm{ij}}$ values were calculated assuming that T = 31.5 days, the average duration between treatment and assessment of the field experiments in the dataset used by Blake et al. (2018).

There was insufficient EC_50_ data to enable parameterisation of $B_{\mathrm{ij}}$ values for a haplotype commonly found with variable numbers of promoter insertions associated with CYP51 overexpression, L50S‐S188N‐I381V‐DEL‐N513K‐CYP↑, which reached high frequencies > 30% in a number of later years included in the analysis, as well as for a small number of other haplotypes with frequencies < 15% in any single year (Table S.3 in Supplementary Material S3). It was therefore necessary to exclude these haplotypes from the analysis.

### 2.4 Fungicide Application Scenarios

For this study we simulated a maximum of two applications of azole fungicides per growing season, in solo or mixture application. We set the timing of the first fungicide application to GS32, that is, $t^{*}$ = 0, and of the second application as $t^{**}$ = 20 days, approximately corresponding to GS39 (when the uppermost leaf of the wheat canopy is fully emerged). We modelled solo application and two‐way, three‐way and four‐way azole mixtures, with the total azole dose kept constant across all scenarios (Table 1). Three‐way and four‐way mixtures are not commonly used in practice, but were included in the analysis to explore the possible benefits of having greater diversity of available active substances.

**TABLE 1:** Example fungicide application scenarios (all possible combinations of the four azoles modelled, for the dose rates presented in this table). 1.0 *j*_1_ denotes a full label dose (maximum permitted individual dose) of azole 1.

Application scenario	Spray 1	Spray 2
Solo	1.0 *j*_1_	1.0 *j*_1_
Two‐way mixture	0.5 *j*_1_ + 0.5 *j*_2_	0.5 *j*_1_ + 0.5 *j*_2_
Three‐way mixture	0.33 *j*_1_ + 0.33 *j*_2_ + 0.33 *j*_3_	0.33 *j*_1_ + 0.33 *j*_2_ + 0.33 *j*_3_
Four‐way mixture	0.25 *j*_1_ + 0.25 *j*_2_ + 0.25 *j*_3_+ 0.25 *j*_4_	0.25 *j*_1_ + 0.25 *j*_2_ + 0.25 *j*_3_+ 0.25 *j*_4_
Case study	*x j*_1_ + (1 − *x*) *j*_2_, 0.05 ≤ *x* ≤ 0.95	*x j*_1_ + (1 − *x*) *j*_2_

All possible combinations of the four azoles tebuconazole, epoxiconazole, prochloraz and prothioconazole were modelled. We modelled additional application rates for a case study of two‐way mixtures of epoxiconazole and prochloraz (using starting haplotype frequencies from 2015), changing the proportion of each azole in the mixture in 0.05 increments between 0.05 *j*_1_ + 0.95 *j*_2_ to 0.95 *j*_1_ + 0.05 *j*_2_. We measured the rate of increase in the epidemic growth rate, $∆\bar{r}$, over 2 years, with the assumption that ${\theta_{i}}_{\text{start}}$ in Year 2 = ${\theta_{i}}_{\mathrm{end}}$ in Year 1 (Equation 15).

### 2.5 Estimating the Impact of the Degree of Cross‐Resistance on Resistance Management Benefits of Azole Mixtures

To investigate to what extent the model predictions of $∆\bar{r}$ for each fungicide programme and starting haplotype frequency (year) scenario can be explained by the degree of cross‐resistance and other explanatory variables, we fitted generalised linear models (‘GLMs’) with a log link function using Genstat statistical software (VSN International 2024) (further details in Supplementary Material S5). The explanatory variables considered were three alternative measures of the degree of cross‐resistance (Section 2.5.1), a measure of average programme efficacy (Section 2.5.2) and a measure of the maximum variance in haplotype sensitivity to any azole component of each mixture (Section 2.5.3). These explanatory variables can be calculated directly from model inputs without a need to run the full model simulation.

#### 2.5.1 Measuring the Degree of Cross‐Resistance

We measured cross‐resistance in a group of haplotypes to two azole fungicides as the frequency‐weighted correlation coefficient of the ED_50_ values expressed as a proportion of the full label field dose, ${D_{\mathrm{ij}}}_{50}$. We denote the weighted correlation coefficient as ${\mathrm{cc}}_{w}$.

(19) $$\begin{array}{c} {\mathrm{cc}}_{w}=\frac{\sum_{i=1}^{M}{\theta_{i}}_{\text{start}}\left({D_{i1}}_{50}-m_{1}\right)\left({D_{i2}}_{50}-m_{2}\right)}{\sqrt{\sum_{i=1}^{M}{\theta_{i}}_{\text{start}}{\left({D_{i1}}_{50}-m_{1}\right)}^{2}}\sum_{i=1}^{M}{\theta_{i}}_{\text{start}}{\left({D_{i2}}_{50}-m_{2}\right)}^{2}} \end{array}$$

where $m_{1}=\sum_{i=1}^{M}{\theta_{i}}_{\text{start}}{D_{i1}}_{50}$ and $m_{2}=\sum_{i=1}^{M}{\theta_{i}}_{\text{start}}{D_{i2}}_{50}$, that is, the frequency‐weighted mean ED_50_ for each azole.

Levels of cross‐resistance between fungicide active substances are typically measured using the unweighted Pearson's correlation coefficient or Spearman's rank correlation coefficient, for example as used by Jørgensen et al. (2021); Kildea et al. (2023, 2024). We denote these measures as ${\mathrm{cc}}_{u}$ and ${\mathrm{cc}}_{z}$ respectively. We tested these measures of correlation between the ED_50_ values of each azole as alternative inputs to the generalised linear model.

In addition to two‐azole cases (Figure 2), we modelled programmes with three and four azoles, for which there are multiple correlation coefficients, one for each combination pair of azoles (e.g., for four azoles there are 6 combination pairs of azoles and thus 6 correlation coefficients). As a simple measure of overall cross‐resistance in the pathogen population to the azoles used in each programme, we used the average of the relevant correlation coefficients.

**FIGURE 2:**
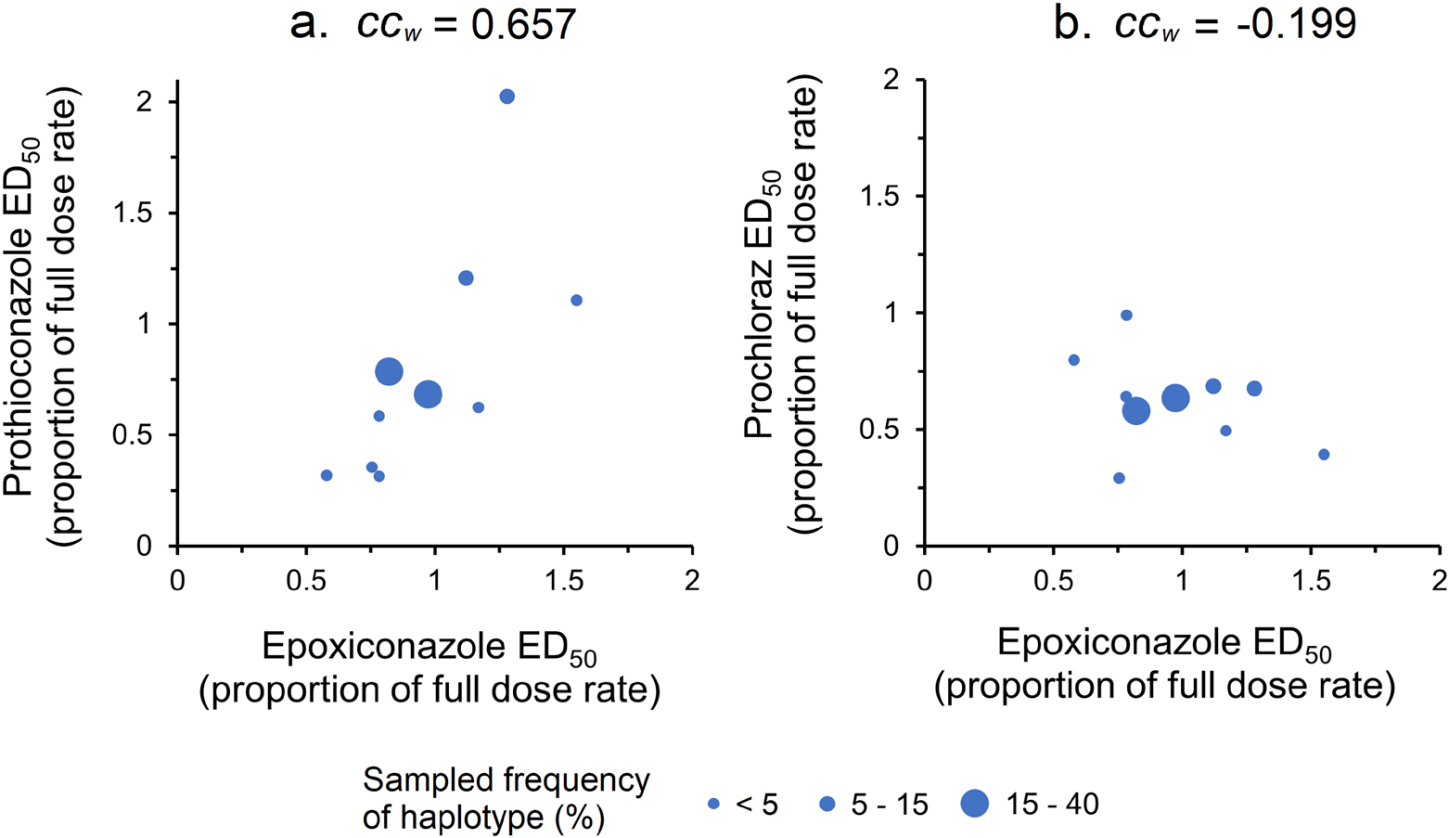
Examples of patterns of cross‐resistance between two azoles, as measured by the weighted correlation coefficient, ${\mathrm{cc}}_{w}$, of the ED_50_ values of CYP51 haplotypes, based on sampled haplotype frequencies in 2015 (*n* = 37). (a) Positive cross‐resistance between epoxiconazole and prothioconazole: ${\mathrm{cc}}_{w}$ = 0.657. (b) Weak negative cross‐resistance between epoxiconazole and prochloraz: ${\mathrm{cc}}_{w}$ = −0.199. Each point on the graph represents a unique CYP51 haplotype. The width of each point represents the initial frequency of the haplotype; note that the displayed widths are not strictly proportional to frequency, to ensure visibility of all points. The sampled frequencies represented are as follows: small points: < 5%; medium points 5%–< 15%; large points: 15%–< 40%.

#### 2.5.2 Average Programme Efficacy

The rate of selection will be greatest when fungicide applications result in a large difference in the growth rates of highly/moderately resistant strains and more sensitive strains. The more effective the fungicide programme against sensitive haplotypes, the larger the potential difference in haplotype growth rates. Therefore, the average efficacy of the azole programme can affect the strength of selection. We measured the average efficacy of each programme as ${\bar{f}}_{\text{start}}$, the average fractional reduction of the pathogen growth rate at the applied dose rate, weighted by the initial haplotype frequencies:

(20) $$\begin{array}{c} {\bar{f}}_{\text{start}}=1-\sum_{i=1}^{M}{\theta_{i}}_{\text{start}}\frac{1}{\sum_{j=1}^{\text{jmax}}B_{\mathrm{ij}}D_{j}+1} \end{array}$$

#### 2.5.3 Variance in Haplotype Sensitivity

The larger the genetic variance in the ED_50_ values of the haplotypes, the greater the potential shift in resistance following application of the azole fungicide (Lande 1979; see Section S.1.4, Supplementary Material S1). For each starting haplotype composition scenario (year), we calculated the variance, ${\mathrm{Var}}_{j}$, in the relative growth rates of each haplotype *i* at the full label field dose of each solo azole *j*, ${D_{j}}_{\max}=1$. Relative growth rates were calculated by setting $r_{0}$ = 1 $t^{-1}$. ${\mathrm{Var}}_{j}$ is then calculated for a population of *M* haplotypes as:

(21) $$\begin{array}{c} {\mathrm{Var}}_{j}=\frac{\sum_{i=1}^{M}{\left(\frac{1}{B_{\mathrm{ij}}+1}-\frac{\sum_{i=1}^{M}\frac{1}{B_{\mathrm{ij}}+1}}{M}\right)}^{2}}{M} \end{array}$$

We used ${\mathrm{Var}}_{j_{\mathrm{Max}}}$, the maximum value of ${\mathrm{Var}}_{j}$ for any azole component in the mixture, as a potential explanatory variable in the generalised linear model.

It should be noted that the variance of the relative growth rates of each haplotype under each mixture application scenario, ${\mathrm{Var}}_{\mathrm{App}}$, could be calculated by replacing the $\frac{1}{B_{\mathrm{ij}}+1}$ terms with $\frac{1}{\sum_{j=1}^{\text{jmax}}B_{\mathrm{ij}}D_{j}+1}$. ${\mathrm{Var}}_{\mathrm{App}}$ is likely to be a useful variable for predicting $∆\bar{r}$, but it is partially dependent on the level of cross‐resistance between azole mixture components. Since the purpose of fitting the generalised linear model was to determine the impact of the degree of cross‐resistance on resistance management benefits of azole mixtures, we did not include ${\mathrm{Var}}_{\mathrm{App}}$ as a potential explanatory variable.

## 3 Results

Table 2 summarises the definitions and values of the fitted model parameters, model variables and generalised linear model variables. The fitted dose response over time for each solo azole product is shown in Figure S.5 (Supplementary Material S2). Model results and generalised linear model inputs for each starting haplotype composition scenario and application scenario (excluding the case study) are given in Table S.6 (Supplementary Material S4).

**TABLE 2:** Definitions of model parameters, model variables and GLM variables.

	Definition	Units	Details	Values
**Parameter**				
$r_{0}$	Average pathogen growth rate without fungicide	$t^{-1}$	Equation (4), Section 2.3.1	0.1173
t	Time: t = 0 corresponds to GS32.	days	Section 2.3.1	—
$t^{*}$	Timing of first azole application (GS32)	days	Section 2.4	0
$t^{**}$	Timing of second azole application (GS39)	days	Section 2.4	20
T	Timing of GS75	days	Section 2.3.1	53
$B_{\mathrm{ij}}$	Dose–response of haplotype *i* to azole *j*	—	Equation (4)	See Table S.5, ^a^
$D_{j0}$	Applied dose of azole *j*	Proportion of full label rate	Equation (5)	See Table 1
α	Decay rate of azole fungicides	$t^{-1}$	Equation (5); Section 2.3.2	0.0924
M	Total number of haplotypes in the population	—	Equation (10)	See Table S.5, ^a^
${\theta_{i}}_{\text{start}}$	Proportion of haplotype i at t = 0	—	Sections 2.2.3 and 2.3.3	See Table S.5, ^a^
$S_{0}$	Initial severity of the total pathogen population at t=0	Proportion of leaf area	Section 2.3.1	4.989 × 10^−4^
$S_{\mathrm{Max}}$	Threshold value of $S\left(T\right)$ for effective disease control	Proportion of leaf area	Section 2.1.2	0.05
**Variable**				
${\bar{r}}_{n}$	Average growth rate of the pathogen population in year *n*	$t^{-1}$	Equation (1); Section 2.2.3	
$∆\bar{r}$	Rate of resistance development	—	Equation (1)	See Table S.6, ^b^
$r_{i}$	Growth rate of haplotype *i*	$t^{-1}$	Equation (4)	
$D_{j}\left(t\right)$	Dose of azole *j* remaining at time *t*	Proportion of full label rate	Equation (5)	
${\theta_{i}}_{\mathrm{end}}$	Proportion of haplotype i at time T	—	Equation (15)	
$S\left(T\right)$	Disease severity at GS75	Proportion of leaf area	Equation (10)	See Table S.6, ^b^
**GLM variable**				
${\mathrm{cc}}_{w}$	Average level of cross‐resistance of haplotypes to two or more azoles	—	Equation (19); Section 2.5.1	See Table S.6, ^b^
${\bar{f}}_{\text{start}}$	Initial (frequency‐weighted) programme efficacy at applied dose rate	Fractional reduction in growth rate	Equation (20)	See Table S.6, ^b^
${\mathrm{Var}}_{j_{\mathrm{Max}}}$	Maximum variance of haplotype sensitivity to a single component of mixture	$t^{-2}$	Section 2.5.3	See Table S.6, ^b^

^a^ Table S.5 (Supplementary Material S3).

^b^ Table S.6 (Supplementary Material S4).

### 3.1 Case Study: Resistance Management Benefit of Mixture Within the Same Mode of Action

A case‐study of two‐way mixtures of epoxiconazole and prochloraz, with starting haplotype frequencies based on a sample taken in 2015 from Harpenden, UK, demonstrates the potential resistance management benefits of mixture within the same mode of action when there is incomplete or negative cross‐resistance. This case study illustrates the model output for one combination of active substances and haplotype frequencies. Outputs from a wider analysis across the parameter space are given later in the results and supplementary material.

In this scenario, there was negative cross‐resistance between epoxiconazole and prochloraz, as ${\mathrm{cc}}_{w}$ = −0.199 (Equation 19, Figure 2). Some haplotypes that were among the most resistant to epoxiconazole were well‐controlled by prochloraz, and vice‐versa (Figure 3, Table S.8, Supplementary Material S5): for example, haplotype L50S‐V136C‐S188N‐A379G‐I381V‐DEL‐S524T was resistant to epoxiconazole but was well‐controlled by prochloraz, and L50S‐V136A‐S188N‐DEL‐S524T was the least sensitive to prochloraz of the haplotypes present, but among the most sensitive to epoxiconazole.

**FIGURE 3:**
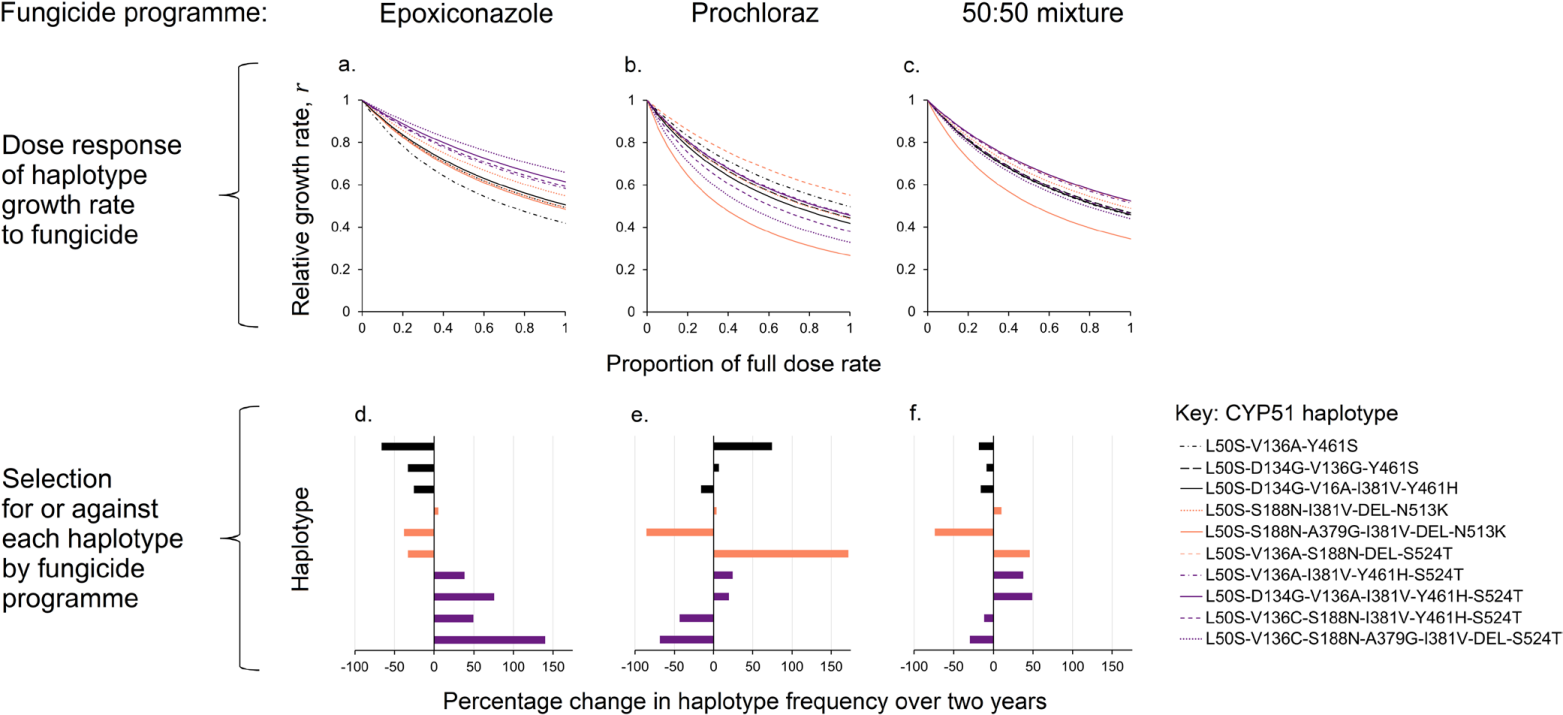
Dose–responses of individual CYP51 haplotypes to (a) epoxiconazole; (b) prochloraz and (c) a 50:50 mixture of epoxiconazole and prochloraz, and the resulting changes in haplotype frequency over 2 years for (d)–(f) epoxiconazole, prochloraz and the 50:50 mixture respectively. We plot relative growth rates by setting $r_{0}$ = 1 $t^{-1}$. Fungicide dose is expressed as a proportion of the full label field dose ${D_{j}}_{\max}$, where the full label field dose for the 50:50 mixture is equal to $0.5{D_{1}}_{\max}+0.5{D_{2}}_{\max}$. Haplotypes shown are those present in 2015 in a sample from Harpenden, UK.

The use of a 50:50 mixture of epoxiconazole and prochloraz reduced the differences in the relative growth rates of the haplotypes, compared with applying epoxiconazole or prochloraz solo (Figure 3), so the selective advantage of the fittest haplotypes was smaller for the mixture. This led to a smaller rate of selection for the 50:50 mixture of epoxiconazole and prochloraz ($∆\bar{r}$ = 0.0071) than for application of solo epoxiconazole ($∆\bar{r}$ = 0.0148) or solo prochloraz ($∆\bar{r}$ = 0.0182) (Table S.8, Supplementary Material S5). The proportion of each azole in the mixture affected both the rate of selection and the level of disease control (Figure S.6, Supplementary Material S5). Comparison of average programme efficacy following selection by the different fungicide programmes suggests that use of azole mixtures could help to protect the efficacy of individual components of the mixture (Table S.8, Supplementary Material S5).

### 3.2 Resistance Management Benefit of Additional Active Substances Within a Mode of Action

#### 3.2.1 Disease Control

In most years, there were several azole mixture programmes incorporating two, three or four azoles that kept disease severity below the 5% threshold denoting effective control, in addition to some solo azole programmes (Figure 4a). From 2016 to 2019, when the average level of azole resistance of the pathogen population was higher than in earlier years, the four‐way mixture did not provide effective disease control, and in 2018 there were no three‐way mixtures that provided effective disease control. In all year scenarios simulated, there were at least 2 two‐way mixtures and at least 1 solo azole programme that achieved effective control.

**FIGURE 4:**
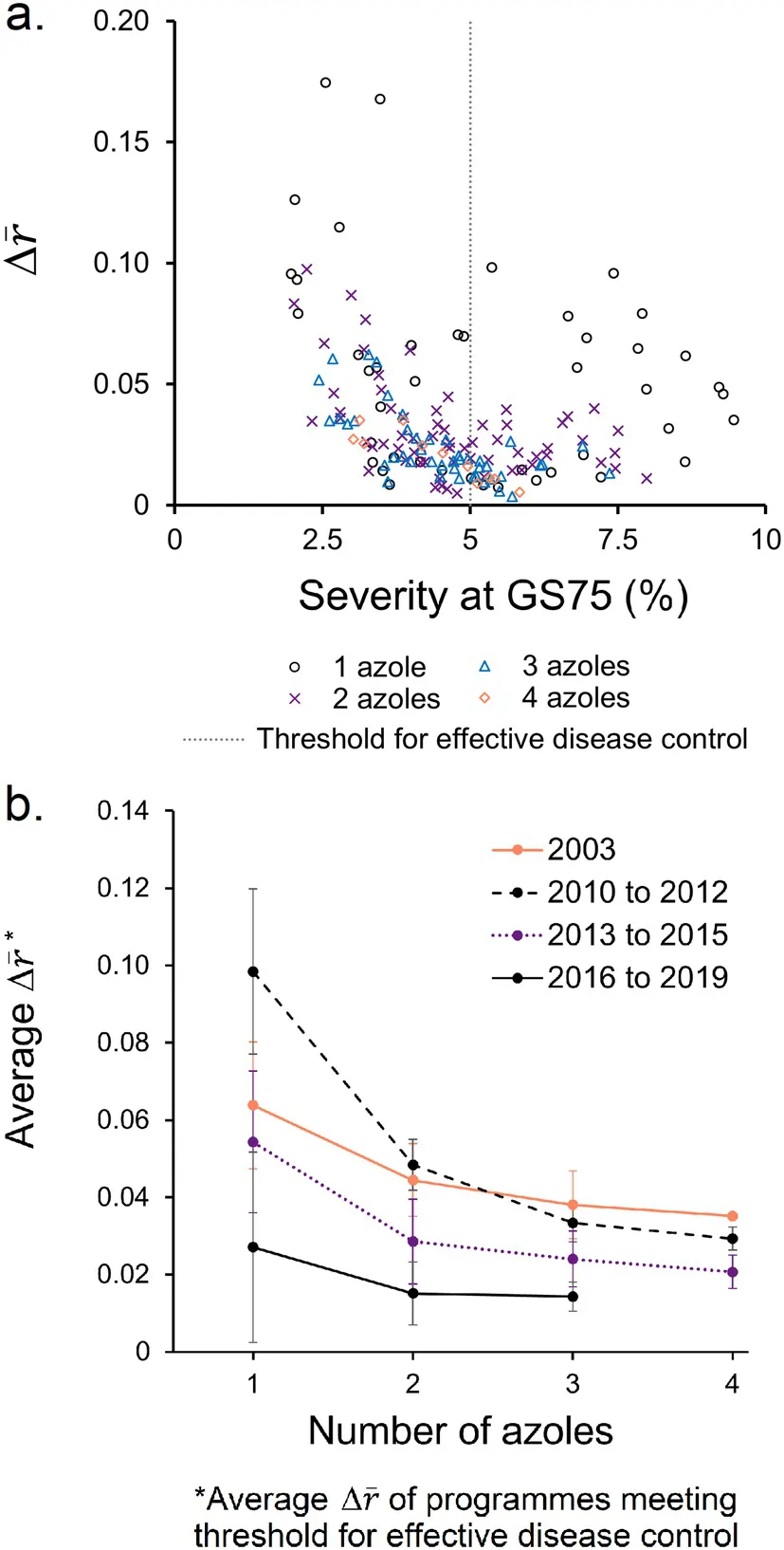
Variation in the rate of resistance development, $∆\bar{r}$, with the number of azoles included in the mixture (‘within‐MoA diversity’). (a) $∆\bar{r}$ over 2 years of application and level of disease control, measured as severity (%) at GS75 in the first year of application for all azole mixture programmes and initial haplotype frequency scenarios (years) modelled. The target for resistance management is programmes that combine effective disease control and a low rate of resistance development, $∆\bar{r}$. Number of azoles in each programme indicated by: 1 azole: black circle; 2 azoles: purple cross; 3 azoles: blue triangle; 4 azoles: orange diamond. The 5% severity threshold for effective disease control is indicated by the grey dotted line. (b) Variation with number of azoles and time of the mean $∆\bar{r}$ of programmes meeting the threshold for effective disease control (< 5% severity at GS75). The mean $∆\bar{r}$ of groups of years is indicated as follows: 2003: orange solid line; 2010–2012: black dashed line; 2013–2015: purple dotted line; 2016–2019: black solid line. Error bars show ±1 standard error of the mean; in cases where only one azole programme in the category met the threshold for effective disease control, these are plotted as points without an error bar. Averages are joined by point‐to‐point lines to aid interpretation.

#### 3.2.2 Selection for Fungicide Resistance

On average, the greater the number of azole active substances included in the mixture, the lower the rate of selection for resistance, as measured by the average $∆\bar{r}$ of programmes meeting the threshold for effective disease control (Figure 4b). The results shown in Figure 4b summarise the predicted rate of selection for solo azoles or azole mixtures over four time periods (2003; 2010–2012; 2013–2015; 2016–2019), where the initial haplotype starting frequency for each year was defined by the frequency of haplotypes sampled in each year (as described in Section 2.3.3).

Results for individual combinations of year and application scenarios are given in Table S.6 (Supplementary Material S4) and illustrated for solo and two‐way mixture programmes in Figure S.7 (Supplementary Material S5). $∆\bar{r}$ was highest in the earlier years simulated (Figure 4b), when there were large differences in growth rates between sensitive and resistant haplotypes. The average level of azole resistance of the population was higher in later years and the differences in growth rates between different resistant haplotypes were smaller, leading to a slower rate of selection for further increases in resistance.

There is evidence in vitro of a secondary mode of action of imidazoles (Elias et al. 2019), which could affect our results for programmes containing prochloraz (see the discussion for further analysis of this point). Figure S.8 (Supplementary Material S5) shows the results presented in Figure 4 with prochloraz excluded from the analysis: the results remain consistent, showing of decreasing average $∆\bar{r}$ as the number of azole active substances included in the mixture increases.

Although there were some solo and two‐way mixture programmes for which $∆\bar{r}$ was very low, the values of $∆\bar{r}$ were more variable for solo and two‐way mixtures, whereas $∆\bar{r}$ was consistently relatively low for three‐way and four‐way mixtures (Figure 5a,b). A well‐chosen combination of two azoles can provide the same or sometimes greater benefit than three‐way or four‐way mixtures. Our results show that availability of a diversity of azole active substances enables mixture programmes that can lower selection.

**FIGURE 5:**
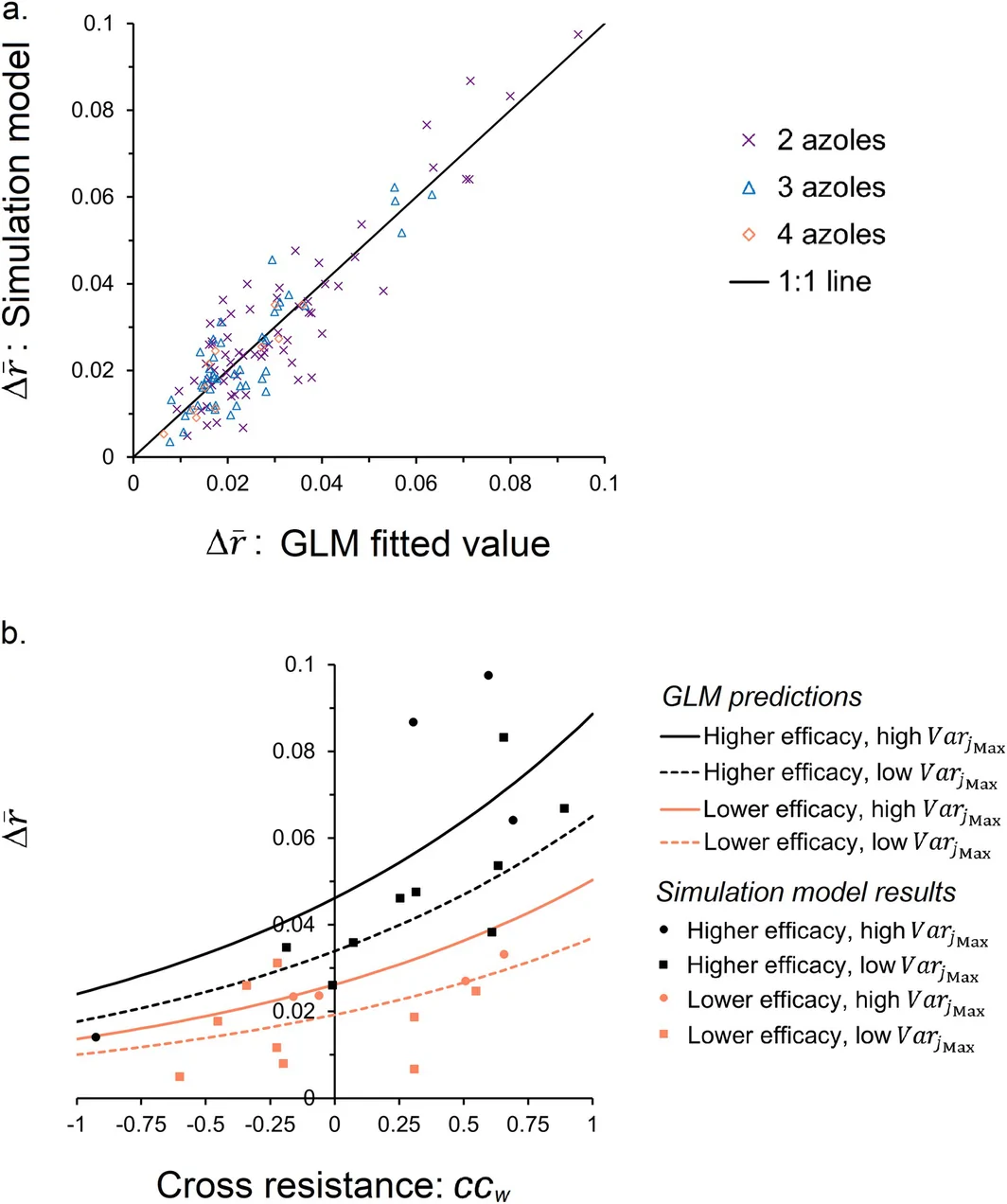
Generalised linear model (GLM) predictions of $∆\bar{r}$ for azole mixtures based on the degree of cross‐resistance between azole components, *𝑐𝑐* ^*w*^, average programme efficacy, ${\bar{f}}_{\text{start}}$, and the maximum variance in haplotype sensitivity for a single azole component of the mixture, ${\mathrm{Var}}_{j_{\mathrm{Max}}}$. (a) Comparison of epidemiological model values vs. GLM fitted values of $∆\bar{r}$ for 2‐, 3‐ and 4‐azole mixtures (denoted as purple cross, blue triangle and orange diamond respectively); solid black line denotes 1:1 line. (b) Variation in $∆\bar{r}$ of 2‐azole mixtures with the degree of cross‐resistance, *𝑐𝑐* ^*w*^, and high and low values of ${\bar{f}}_{\text{start}}$ and ${\mathrm{Var}}_{j_{\mathrm{Max}}}$. The higher and lower values of ${\bar{f}}_{\text{start}}$ correspond to final disease severity at GS75 of 2%–3.5% and 4.5%–5.5% respectively. GLM predictions are denoted as follows for the different levels of ${\bar{f}}_{\text{start}}$ (higher = 0.62; lower = 0.51) and ${\mathrm{Var}}_{j_{\mathrm{Max}}}$ (high = 0.019 $t^{-2}$; low = 0.0075 $t^{-2}$): high efficacy and high ${\mathrm{Var}}_{j_{\mathrm{Max}}}$: solid black line; high efficacy and low ${\mathrm{Var}}_{j_{\mathrm{Max}}}$: dashed black line; lower efficacy and high ${\mathrm{Var}}_{j_{\mathrm{Max}}}$: solid orange line; lower efficacy and low ${\mathrm{Var}}_{j_{\mathrm{Max}}}$. Epidemiological model results for similar categories of ${\bar{f}}_{\text{start}}$ (higher: 0.585 < ${\bar{f}}_{\text{start}}$ < 0.687; lower: 0.485 < ${\bar{f}}_{\text{start}}$ < 0.526) and ${\mathrm{Var}}_{j_{\mathrm{Max}}}$ (high: 0.0165 $t^{-2}$ < ${\mathrm{Var}}_{j_{\mathrm{Max}}}$ < 0.0275 $t^{-2}$; low: 0.0021 $t^{-2}$ < ${\mathrm{Var}}_{j_{\mathrm{Max}}}$ < 0.0118 $t^{-2}$) are denoted as follows: high efficacy and high ${\mathrm{Var}}_{j_{\mathrm{Max}}}$: black circle; high efficacy and low ${\mathrm{Var}}_{j_{\mathrm{Max}}}$: black square; low efficacy and high ${\mathrm{Var}}_{j_{\mathrm{Max}}}$: pink circle; low efficacy and low ${\mathrm{Var}}_{j_{\mathrm{Max}}}$: pink square.

### 3.3 Impact of the Degree of Cross‐Resistance on Resistance Management Benefits of Azole Mixtures

Comparing the three measures of cross‐resistance, the frequency‐weighted correlation coefficient of the ED_50_ values, ${\mathrm{cc}}_{w}$ (defined in Equation 19), was the best predictor for the rate of selection for resistance, $∆\bar{r}$ (Table S.10, Supplementary Material S5). As there were significant interactions between the number of azoles and the fitted coefficients (see Tables S.11–S.13 in Supplementary Material S5), we fitted individual GLMs of $∆\bar{r}$ for solo azoles, two‐way, three‐way and four‐way mixtures (Table 3, Tables S.14–S.17 in Supplementary Material S5). The GLMs using ${\mathrm{cc}}_{w}$ as the measure of cross‐resistance achieved adjusted R^2^ values of 83.0%, 84.0% and 88.2% respectively for 2‐, 3‐ and 4‐azole programmes (Figure 5a, Table 3). The degree of cross‐resistance, ${\mathrm{cc}}_{w}$ explained 36.6%, 38.6% and 36.0% of the variance in $∆\bar{r}$ respectively for 2‐, 3‐ and 4‐azole programmes (Table 3). The cross‐resistance of an azole with itself is always equal to 1: a GLM fitted for solo azole programmes only, using ${\mathrm{Var}}_{j_{\mathrm{Max}}}$ and ${\bar{f}}_{\text{start}}$ as explanatory variables, achieved an adjusted R^2^ of 80.1% (Table 3, Figure S.9, Supplementary Material S5).

**TABLE 3:** Fitted parameter values for generalised linear models with a log link function for 1, 2, 3 and 4‐azole programmes.

Number of azoles	1	2	3	4	1	2	3	4
Parameter	Estimate (SE)				% variance explained by variate in GLM			
Intercept	−6.545 (0.384)	−7.213 (0.280)	−8.702 (0.440)	−9.799 (0.943)	—	—	—	—
*𝑐𝑐* ^*w*^	N/A	0.697 (0.084)	0.774 (0.125)	1.769 (0.689)	—	36.6	38.6	36.0
${\bar{f}}_{\text{start}}$	5.353 (0.602)	5.835 (0.456)	8.137 (0.720)	9.32 (1.49)	36.4	35.0	40.2	48.7
${\mathrm{Var}}_{j_{\mathrm{Max}}}$	63.96 (5.43)	28.00 (4.03)	22.86 (5.27)	31.3 (11.8)	44.6	12.1	6.4	7.1

$∆\bar{r}$ was predicted to increase with increasing levels of cross‐resistance, ${\mathrm{cc}}_{w}$, average programme efficacy, ${\bar{f}}_{\text{start}}$, and maximum variance in haplotype sensitivity to a single azole component of the mixture, ${\mathrm{Var}}_{j_{\mathrm{Max}}}$ (Table 3, Figure 5b). Figure 5b shows the variation in $∆\bar{r}$ predicted by the general linear model for 2‐azole mixtures with varying levels of cross‐resistance, programme efficacy and maximum variance in haplotype sensitivity. For programmes with a combination of high programme efficacy and high ${\mathrm{Var}}_{j_{\mathrm{Max}}}$, there is a steep decrease in $∆\bar{r}$ as the degree of cross‐resistance between azoles in the mixture decreases, although in these cases $∆\bar{r}$ is still relatively high even for mixtures of azoles with negative cross‐resistance. Relatively low values of ${\bar{f}}_{\text{start}}$ and ${\mathrm{Var}}_{j_{\mathrm{Max}}}$ are associated with lower values of $∆\bar{r}$, but in these cases there is a shallower gradient between ${\mathrm{cc}}_{w}$ and $∆\bar{r}$: the rate of selection is still minimised by low or negative cross‐resistance, but the predicted change in $∆\bar{r}$ is smaller.

Several combinations of azole active substances consistently minimised or maximised $∆\bar{r}$ for several years in a row, in addition to meeting the threshold for effective disease control (Table S.9, Supplementary Material S5). This suggests that some azole mixtures can provide resistance management benefits, despite variation in the values of ${\mathrm{cc}}_{w}$, ${\bar{f}}_{\text{start}}$ and ${\mathrm{Var}}_{j_{\mathrm{Max}}}$ for each azole programme between each year due to changes in haplotype composition. On average, azole mixtures that consistently minimised $∆\bar{r}$ had negative or low levels of positive cross‐resistance between azole components, and relatively low levels of programme efficacy; close to the threshold for effective disease control.

## 4 Discussion

We have described a novel method for modelling the joint action of active substances within a MoA with incomplete cross‐resistance, where the dose response can be parameterised based on measured lab EC_50_ values. The method was then applied to the case of CYP51 haplotypes defining resistance phenotypes to azole fungicides, using the rate of change in the pathogen population growth rate in fungicide‐treated crops, $∆\bar{r}$, as a practical measure for assessing the effects of selection for resistance. Our results show that there can be resistance management benefits of mixtures within a MoA, particularly where there is low or negative cross‐resistance between the active substances in the mixture. Based on observed haplotype frequencies, we show that use of azole mixtures could, in principle, help to protect the efficacy of the individual components of the mixture and provide a more stable level of control than solo application. The analysis focused on within‐MoA mixtures, but it is important to note that, in practice, azoles should be used in combination with other MoAs when possible.

The results show that greater diversity of active substances within a MoA can be helpful to enable use of mixtures that reduce selection. Some two‐way mixtures minimised selection, but some did not. This finding is consistent with the results of both Dooley et al. (2016), where no resistance management benefits were seen from the mixture of two azoles with relatively strong positive cross‐resistance, and those of Heick et al. (2017), who showed that more diverse azole programmes reduced selection overall for CYP51 alterations. The resistance management benefits of active substance diversity within a MoA are likely to be greater for fungicides with low cross‐resistance with other active substances and a similar level of efficacy. The analysis focused on azole products with a long history of use; hence the efficacy of some active substances had already been substantially eroded. The findings are also of relevance for the newer azole mefentrifluconazole which has low cross‐resistance with prothioconazole (Heick et al. 2020; Kildea et al. 2024).

Our results show a trade‐off between efficacy and selection, as noted in previous studies (including Corkley et al. 2025; Hall et al. 2004; Taylor and Cunniffe 2023a). The rate of selection for resistance is increased by higher programme efficacy and higher variability in the fitness of haplotypes against mixture components. This trade‐off increases the challenge of resistance management for the most effective fungicides, as fungicide programmes which are very effective against sensitive haplotypes can increase the relative fitness of resistant haplotypes and lead to faster selection for resistance. Accepting fungicide programmes with efficacy closer to the threshold for effective disease control could therefore delay the development of resistance. However, if azole fungicides are being used in mixture with other single‐site acting MoA (which are themselves at risk of resistance), it may be necessary to choose azole active substances which do not minimise $∆\bar{r}$ for azole programmes alone, to ensure that the efficacy of both mixture partners is balanced. Broad‐spectrum control against a range of plant pathogens and fungicide cost will also drive farmers' decision‐making around choice of azoles. Our approach could help to identify combinations of azole active substances that can provide resistance management benefits in combination with effective disease control. Monitoring to detect any shifts in patterns of cross‐resistance within the MoA could support timely adaptation of resistance management tactics as required.

We assumed that all azoles included in this study had the same decay rate, as there were no significant differences in mean half‐lives based on estimates available in the public domain; this assumption reduced the computational intensity of the modelling analysis. If the decay rate differs between azoles, azoles with longer half‐lives could be left acting effectively solo after the effect of other azoles in the mixture has ended (Corkley et al. 2022). This would likely result in relatively stronger selection for haplotypes with resistance to the azole with the slower decay rate. This could be at least partially compensated for by choice of a different ratio of azole products in the mixture. High‐quality data providing direct comparison of azole foliar dissipation half‐lives under a range of conditions would therefore be useful in design of azole mixture products or programmes.

We assumed that inhibition of the CYP51 enzyme was the sole mode of action of all the azoles modelled: experimental evidence shows that triazoles do not inhibit mutant strains that lack the CYP51 enzyme (Elias et al. 2019). However, there is in vitro evidence that imidazoles have a secondary or multimodal mode of action, particularly at high concentrations (Sud and Feingold 1981): imidazoles inhibit mutant strains lacking the CYP51 enzyme (Elias et al. 2019). The secondary mode of action may relate to non‐selective inhibition of other heme‐containing monooxygenases in fungal cells, or specifically inhibition of membrane‐bound enzymes and membrane lipid biosynthesis (Elias et al. 2019; Ghannoum and Rice 1999). The balance of activity in vivo between the CYP51 inhibition and secondary modes of action is unknown, but the existence of a secondary mode of action could impact on our results for prochloraz. The assumption that the prochloraz acts solely on the CYP51 enzyme and that there is no independent action against other target sites could lead to conservative estimates of the resistance management benefit of including prochloraz in an azole‐mixture programme. Conversely, the inclusion of prochloraz in the analysis could overstate the effects of cross‐resistance occurring purely within a single mode of action. However, Figure S.8 shows that the general trend of our results remains consistent even when prochloraz is excluded from the analysis.

We used observed haplotype frequencies to inform the starting points for population composition in the scenarios modelled. The analysis was restricted to the effect of selection on haplotypes that were already present and did not estimate the likelihood of the emergence of novel haplotypes over the course of the two‐year period simulated. The repeatability of CYP51 mutations in the field is low (Hawkins and Fraaije 2021), so it is very difficult to predict which new haplotypes might emerge in response to different azole programmes. However, selective pressures on emerging strains could be affected by the use of azole mixtures, potentially selecting for different haplotypes that were not seen in the sampled dataset. Overreliance on within‐MoA diversity and incomplete cross‐resistance for resistance management could increase selection for generalised mechanisms of azole resistance, as noted by Dooley et al. (2016). For example, target‐site overexpression can reduce sensitivity to all azole fungicides, whilst non‐target‐site resistance mechanisms, such as the overexpression of efflux pumps, are a risk both as a source of generalised azole resistance and for positive cross‐resistance with other MoAs (Cools et al. 2013). The model could potentially be adapted to explore the effects of target‐site overexpression and non‐target‐site resistance in combination with the target‐site mutations modelled in this analysis. This would require suitable data on the frequency and heritability of different levels of CYP51 overexpression and efflux mechanisms in combination with CYP51 haplotype and model representation of the effects of sexual reproduction.

A small number of haplotypes that appeared in the samples could not be included in the analysis due to insufficient EC_50_ data; most of these were only found at low frequencies and so would probably have had minimal impact on the overall value of $∆\bar{r}$ calculated for the two‐year period simulated. One haplotype that could not be included—L50S‐S188N‐I381V‐DEL‐N513K‐CYP↑, which is associated with variable levels of CYP51 overexpression and sensitivity—was found at higher frequencies in the later years included in the analysis. Since CYP51 overexpression is associated with generalised azole resistance (Cools et al. 2013), the exclusion of this haplotype may lead to overestimation of the benefits of azole mixtures in these years. As discussed above, further data would be required to adequately represent this case. Some individual haplotype EC_50_ values were larger than the average population EC_50_ used by Blake et al. (2018) to calculate EC_50_ to ED_50_ values, requiring some extrapolation beyond the fitted relationship. Our estimates of $B_{\mathrm{ij}}$ are therefore more uncertain for some highly resistant haplotypes present in the later years simulated.

We assumed that there was no fitness cost of resistance for the haplotypes included in the analysis. All haplotypes included in the analysis reached a minimum frequency > 2% in the field samples; substantial fitness costs can occur in strains selected in the field, but appear to be relatively rare, or sometimes transitory due to compensatory mutations (Hawkins and Fraaije 2018; Mikaberidze et al. 2014). Data measuring the impact of fitness costs on different parts of the life cycle and in varying environmental conditions would be needed to accurately represent fitness costs for individual haplotypes. If fitness costs were present for some haplotypes, this would lower their competitive fitness relative to other haplotypes and so could affect our results for individual years where those haplotypes were present. However, it is very unlikely that this would change our qualitative finding of resistance management benefits of mixtures within a MoA where there is low or negative cross‐resistance between the active substances in the mixture.

Our analysis covers the example of *Z. tritici* infection of wheat crops, but our results are highly relevant for other plant pathogens with analogous patterns of incomplete cross‐resistance to azoles driven by differing CYP51 haplotypes, for example as reported for *P. pachyrhizi* and *C. beticola* (Kayamori et al. 2021; Stilgenbauer et al. 2023) which are also polycyclic foliar pathogens with similar life cycles to *Z. tritici*. Synergistic effects of azole mixtures have also been reported in *Fusarium graminearum* (the cause of Fusarium head blight (FHB) in cereal crops), which has paralagous CYP51 genes, with *CYP51C* mediating differential sensitivity to two triazoles (Liu et al. 2011). Our approach and results may also have broader applications beyond agriculture, since there is also evidence of incomplete cross‐resistance between azoles used in medicine, for example in *Candida* spp. and *Cryptococcus neoformans* (Illnait‐Zaragozi et al. 2008; Li et al. 2025). We are not aware of any studies considering how azole combination therapy in medicine affects selection for resistance; the potential impact on the rate and severity of side effects in the human body would also need to be assessed.

There is potential for incomplete cross‐resistance in other fungicide MoAs to prove useful for resistance management: for example, there are varying levels of positive cross‐resistance between SDHI fungicides in *Z. tritici* and in other pathogens including *Botrytis cinerea* (gray mould pathogen which significantly impacts multiple crops), *Alternaria alternata* (the cause of Alternaria leaf spot), *Corynespora cassiicola* (the cause of Corynespora leaf spot) and *Podosphaera xanthii* (the cause of cucurbit powdery mildew) (Avenot et al. 2016; Hawkins and Fraaije 2021; Miyamoto et al. 2020; Peng et al. 2021; Veloukas et al. 2013; Zhu et al. 2022). The method introduced for modelling the joint target‐site action of multiple active substances within a MoA is likely to be applicable to other MoA, although inheritance of resistance phenotype may be more complex if it is not confined to a single target‐site gene and so the model may require adaptation for cases other than azole fungicides. Resistance to succinate dehydrogenase inhibitor (SDHI) fungicides can occur due to target‐site mutations on the *SDHB*, *SDHC* and *SDHD* genes which code for subunits of the succinate dehydrogenase enzyme, and in some isolates due to the presence of an additional SDHC paralog, named alt‐SDHC (Steinhauer et al. 2019; Yamashita and Fraaije 2018). The effect of analogous mutations on cross‐resistance to SDHIs varies by species, possibly due to epistatic interactions (Hawkins and Fraaije 2021; Peng et al. 2021). To model resistance evolution following application of SDHI mixtures in pathogens with a sexual stage, it would be necessary to include the effect of sexual reproduction, with phenotype depending on the combination of mutations on more than one SDH‐ subunit and/or presence of additional SDHC paralogs (Gutiérrez‐Alonso et al. 2017; Sun et al. 2022).

Between‐MoA mixtures often provide strong resistance management benefits because of a general lack of cross‐resistance between mixture partners. Our results show that within‐MoA mixtures can also be useful for fungicide resistance management where there is low or negative cross‐resistance between mixture partners. This insight suggests that within‐MoA mixtures could provide additional flexibility in the selection of resistance management tactics, especially in cases where there are limited options for mixture between MoAs.

## Funding

The authors thank BASF plc for project funding (project 1020280). I.C. acknowledges AHDB for PhD Studentship funding (project 21120062). B.A.F. and N.J.H. acknowledge AHDB for project (PR475 and PR619) funding. Rothamsted Research receives strategic funding from the Biotechnology and Biological Sciences Research Council of the United Kingdom. A.E.M. and J.H. acknowledge support from the Growing Health Institute Strategic Programme (BBS/E/RH/230003C).

## Conflicts of Interest

The authors declare no conflicts of interest.

## Supporting information


**Supplementary Material S1** Further details on the population genetic model.
**Figure S.1** The process of converting lanosterol into ergosterol, top row, and competitive binding and release of two azole fungicides to the CYP51 enzyme.
**Figure S.2** Ergosterol production rate, *V*, as a function of the total azole dose, D, of two azoles in a 1:1 ratio ($D_{1}=$ $D_{2}$), for a range of values of dose response parameter $B_{1}$ and $B_{2}=1$.

**Supplementary Material S2** Further details on model parameterisation.
**Table S.1** Estimates of foliar dissipation half‐life (‘DT50’) of tebuconazole, epoxiconazole, prochloraz and prothioconazole‐desthio available in public domain sources.
**Figure S.3** Relative frequencies of CYP51 haplotype frequencies for which in vitro EC_50_ dose–response measurements were available, as sampled from the *Zymoseptoria tritici* population in fields around Rothamsted Research, Harpenden, UK, in 2010 to 2019. Haplotypes that reached a minimum frequency of 0.15 in one or more years from 2010 to 2019 shown.
**Figure S.4** Fitted relationship between log(prothioconazole EC_50_) (log (μg/mL)) and log(prothioconazole‐desthio EC_50_) (log (μg/mL)). Adjusted *R* ^2^ = 63.95%.
**Figure S.5** Mean fitted dose response over time for epoxiconazole, prochloraz, prothioconazole and tebuconazole.

**Supplementary Material S3:** Datasets used for model parameterisation.
**Table S.2** Dataset metadata for Tables S3–S5.
**Table S.3** Relative haplotype frequencies as sampled in Harpenden, UK in the years 2003 and 2010–2019.
**Table S.4** Isolates of each haplotype used to estimate mean EC50 values for each haplotype‐azole combination.
**Table S.5** Relative haplotype frequencies and dose response parameters *B*_*ij*_ used in modelling scenarios, and the mean EC50 values for each haplotype used to derive the field dose *D*_*ij*_50 and dose response parameters.
**Supplementary Material S4:** Table of model results and generalized linear model inputs.

**Table S.6** Model results and generalised linear model inputs for each combination of solo or mixture application scenario and starting haplotype composition scenario.

**Supplementary Material S5** Further details on model results.
**Table S.7** Comparison of $∆\bar{r}$ and ED_50_ shift as measures of the development of resistance, for a number of example cases covering a range from low to high $∆\bar{r}$ values and small to large ED_50_ shifts.
**Figure S.6** Variation in the rate of resistance development, $∆\bar{r}$, and epidemic severity with the ratio of two azoles in a mixture: prochloraz and epoxiconazole, for a *Z. tritici* population with the CYP51 haplotype frequencies sampled in 2015 in Harpenden, UK.
**Table S.8** Comparison of the effects of 2 years of application of epoxiconazole (solo), prochloraz (solo) or a 50:50 mixture of epoxiconazole and prochloraz on selection of individual CYP51 haplotypes, and the overall resulting change in fungicide programme efficacy, for initial haplotype frequencies based on a sample from 2015 from Harpenden, UK.
**Table S.9** Azole programmes meeting the threshold for effective control that tended to minimise or maximise $∆\bar{r}$ most frequently for the scenarios (i.e., sampled haplotype starting frequencies by year) modelled, and range of values of cross‐resistance (*cc*_*w*_), programme efficacy (${\bar{f}}_{\text{start}}$) and maximum variance in haplotype sensitivity (${\mathrm{Var}}_{j_{\mathrm{Max}}}$) over the years relevant for each programme.
**Figure S.7** Variation in model results for solo and two‐way mixture azole programmes over time.
**Figure S.8** Variation in the rate of resistance development, $∆\bar{r}$, with the number of azoles included in the mixture (‘within‐MoA diversity’), excluding prochloraz from the analysis (for comparison with the results presented in Figure 4).
**Table S.10** Percentage variance in $∆\bar{r}$ explained by individual variates included in generalised linear models (GLMs) with a log link function for 1, 2, 3 and 4‐azole programmes.
**Table S.11** Percentage variance in $∆\bar{r}$ of 1, 2, 3 and 4‐azole programmes explained by individual variables in generalised linear models (GLMs) with a log‐link function, with only one explanatory variable per GLM.
**Table S.12** Accumulated analysis of variance for a generalised linear model of $∆\bar{r}$ with a log link function for two and three‐azole mixture programmes.
**Table S.13** Accumulated analysis of variance for a generalised linear model of $∆\bar{r}$ with a log link function for 2‐azole mixture programmes and solo azole programmes.
**Table S.14** Accumulated analysis of variance for a generalised linear model of $∆\bar{r}$ with a log link function for solo azole programmes.
**Table S.15** Accumulated analysis of variance for a generalised linear model of $∆\bar{r}$ with a log link function for two‐azole mixture programmes.
**Table S.16** Accumulated analysis of variance for a generalised linear model of $∆\bar{r}$ with a log link function for three‐azole mixture programmes.
**Table S.17** Accumulated analysis of variance for a generalised linear model of $∆\bar{r}$ with a log link function for four‐azole mixture programmes.
**Figure S.9** Comparison of epidemiological model values vs. GLM fitted values of $∆\bar{r}$ for solo azole applications (denoted as black circle); solid black line denotes 1:1 line.

## Data Availability

The data that support the findings of this study are available in the Supplementary Material of this article.
