# Supplementary material for: Modelling Resistance Management Benefits of Incomplete Cross‐Resistance Within a Fungicide Mode of Action Group
Source: Evol Appl. 2026 Nov 5;19(11):e70346. doi: 10.1111/eva.70346 (PMC13736046; doi:10.1111/eva.70346)
Supplement: Supplementary file 1 [file EVA-19-e70346-s001.docx]

**Supplementary Material S1: Further details on the population genetic model**

S.1.1. Further details on modelling the azole dose-response curve

Figure S.1: The process of converting lanosterol into ergosterol, top row, and competitive binding and release of two azole fungicides to the CYP51 enzyme. Enzyme, E, blue. Lanosterol molecule, L, yellow. Ergosterol molecule, E_r_, green. Azole 1 molecule, F_1,_ red. Azole 2 molecule, F_2,_ purple. *k_a_* is the rate of binding and k_-a_ is the unbinding rate for each process. Rate *k_2_* denotes the combined rate of conversion of lanosterol to ergosterol and unbinding of the product from the enzyme.

Figure S.2: Ergosterol production rate, *V*, as a function of the total azole dose, $D$, of two azoles in a 1:1 ratio ($D_{1}=$ $D_{2}$), for a range of values of dose response parameter $B_{1}$ and $B_{2}=1$. We plot relative rates by setting $A$ =1$t^{-1}$. See Equation 3, Section 2.2.1.

*Assumption that azoles bind competitively to the CYP51 enzyme*

In the derivation of the dose-response curve we have assumed that azole molecules bind competitively to the CYP51 enzyme. There is an ongoing debate about the binding dynamics of azoles to the CYP51 molecule. We thank Dr Marcus Fehr for his insights into the debate. Several authors say that azoles bind non-competitively to the CYP51 molecule (e.g. Parker et al., 2011). They make this claim because of the observed effect of the substrate concentration on the rate of product development. This is however an indirect observation. Direct observations on the binding dynamics, such as those carried out by Hargrove et al. (2018), suggest that binding is of the competitive type.

Using competitive inhibition or non-competitive inhibition does affect the rate of product formation as dependent on the substrate concentration. However, to model the dose-response the substrate concentration is not relevant, only the effect of inhibitor concentration.

Wang (2014) derived both cases step by step and the reader is referred to those lecture notes. In his notation, which is slightly different from ours, Wang shows that the rate of product formation for the case of competitive binding is given by:

$$\begin{aligned} V=\frac{k_{2}E_{0}S}{K_{m}\left( 1+\frac{I}{K_{I}} \right)+S}\#\left( S.1 \right) \end{aligned}$$

And in the case of non-competitive binding by:

$$\begin{aligned} V=\frac{k_{2}E_{0}S}{\left( 1+\frac{I}{K_{I}} \right)\left( K_{m}+S \right)}\#\left( S.2 \right) \end{aligned}$$

where $S$ is the substrate concentration and $I$ is the inhibitor concentration, and the rest of the symbols are other parameters. It is clear from Equations (A.1) and (A.2) that the effect of substrate concentration on product formation is different, but also that the effect of the inhibition concentration on product formation is the same as both equations can be written as $V=\frac{A}{1+BI}$, which is the form of our dose-response curve (Equation 2, main paper).

S.1.2 Solving the model for $S_{i}\left( T \right)$

The dose dynamics of a fungicide spray, dose response curve, and dynamics of disease severity (lesion density) are described by Equations (6), (7) and (6) and (8), and the season length is defined as $T$. (Section 2.2.2; Section 2.2.2.1, main paper). We can use these to derive equations for the severity of haplotype $i$ at time $T$, $S_{i}\left( T \right)$, after one or two applications of fungicide $j$.

Consider one application of dose $D_{j0}$ at $t=0$:

$$\begin{aligned} \frac{dS_{i}\left( t \right)}{dt}=\frac{r_{0}}{1+B_{ij}D_{j0}e^{-\alpha t}}S_{i}\left( t \right) \to\frac{1}{S_{i}\left( t \right)}\frac{dS_{i}\left( t \right)}{dt}=\frac{r_{0}}{1+B_{ij}D_{j0}e^{-\alpha t}}\#\left( S.3 \right) \end{aligned}$$

Integrating with respect to time from $t=0$ to $t=T$:

$\begin{aligned} \int_{0}^{T} \frac{1}{S_{i}\left( t \right)}dS_{i}\left( t \right)=\int_{0}^{T} \frac{r_{0}}{1+B_{ij}D_{j0}e^{-\alpha t}}dt\to\ln\left( S_{i}\left( t \right) \right)-\ln\left( S_{i}\left( 0 \right) \right)=\int_{0}^{T} \frac{r_{0}}{{1+B}_{ij}D_{j0}e^{-\alpha t}}dt= \frac{r_{0}}{\alpha}\int_{0}^{T} \frac{\alpha e^{\alpha t}}{B_{ij}D_{j0}+e^{\alpha t}}dt \#\left( S.4 \right) \end{aligned}$

To solve the integral at the right-hand side, we define $u=B_{ij}D_{j0}+e^{\alpha t}$. Then $u^{'}=\alpha e^{\alpha t}$.

$$\begin{aligned} \frac{r_{0}}{\alpha}\int_{0}^{T} \frac{\alpha e^{\alpha t}}{B_{ij}D_{j0}+e^{\alpha t}}dt=\frac{r_{0}}{\alpha}\int_{0}^{T} \frac{u^{'}}{u}dt=\frac{r_{0}}{\alpha}\int_{0}^{T} \frac{1}{u}du=\frac{r_{0}}{\alpha}\ln\left( u \right)\left| \begin{matrix} T \\ 0 \end{matrix} \right.=\frac{r_{0}}{\alpha}\ln\left( B_{ij}D_{j0}+e^{\alpha t} \right)\left| \begin{matrix} T \\ 0 \end{matrix} \right. \#\left( S.5 \right) \end{aligned}$$

Therefore:

$$\begin{aligned} \frac{r_{0}}{\alpha}\int_{0}^{T} \frac{\alpha e^{\alpha t}}{B_{ij}D_{j0}+e^{\alpha t}}dt=\frac{r_{0}}{\alpha}\ln\left( B_{ij}D_{j0}+e^{\alpha t} \right)-\frac{r_{0}}{\alpha}\ln\left( B_{ij}D_{j0}+1 \right)\#\left( S.6 \right) \end{aligned}$$

Substituting $S_{i}\left( t \right)$ into equation (A.6) gives:

$$\begin{aligned} \ln\left( S_{i}\left( t \right) \right)-\ln\left( S_{i}\left( 0 \right) \right)=\frac{r_{0}}{\alpha}\ln\left( B_{ij}D_{j0}+e^{\alpha t} \right)-\frac{r_{0}}{\alpha}\ln\left( B_{ij}D_{j0}+1 \right)\#\left( S.7 \right) \end{aligned}$$

$$\begin{aligned} S_{i}\left( t \right)=S_{i}\left( 0 \right)\left( \frac{B_{ij}D_{j0}+e^{\alpha t}}{B_{ij}D_{j0}+1} \right)^{\frac{r_{0}}{\alpha}}\#\left( S.8 \right) \end{aligned}$$

If there is a time period $t^{*}$ between $t=0$ and the spray application, and the season length is $t=T$, then:

$$\begin{aligned} S_{i}\left( T \right)=S_{i}\left( t^{*} \right)\left( \frac{B_{ij}D_{j0}+e^{\alpha\left( T-t^{*} \right)}}{B_{ij}D_{j0}+1} \right)^{\frac{r_{0}}{\alpha}}\#\left( S.9 \right) \end{aligned}$$

Since $S_{i}\left( t^{*} \right)=S_{i}\left( 0 \right)e^{r_{0}t^{*}}$, we find Equation 7 (main paper):

$$S_{i}\left( T \right)=S_{i}\left( 0 \right)e^{r_{0}t^{*}}\left( \frac{B_{ij}D_{j0}+e^{\alpha\left( T-t^{*} \right)}}{B_{ij}D_{j0}+1} \right)^{\frac{r_{0}}{\alpha}}$$

Consider two applications, one at $t^{*}$ and one at $t^{**}$. First it is necessary to calculate what dose remains at time $t^{**}$ from the first application at time $t^{*}$, following dose decay over the time period $t^{**}-t^{*}$; this dose is added to the application dose at $t^{**}$, $D_{j0}$:

$$\begin{aligned} D\left( t^{**} \right)=D_{j0}e^{-\alpha\left( t^{**}-t^{*} \right)}+D_{j0} \#\left( S.10 \right) \end{aligned}$$

Therefore for a two-spray programme, we find Equation 8 (main paper):

$$S_{i}\left( T \right)=S_{i}\left( 0 \right)e^{r_{0}t^{*}}\left( \frac{B_{ij}D_{j0}+e^{\alpha\left( t^{**}-t^{*} \right)}}{B_{ij}D_{j0}+1} \right)^{\frac{r_{0}}{\alpha}}\left( \frac{B_{ij}D_{j0}\left( 1+e^{-\alpha\left( t^{**}-t^{*} \right)} \right)+e^{\alpha\left( T-t^{**} \right)}}{B_{ij}D_{j0}\left( 1+e^{-\alpha\left( t^{**}-t^{*} \right)} \right)+1} \right)^{\frac{r_{0}}{\alpha}}$$

S.1.3. The instantaneous average growth rate

As noted in Section 2.2.3, $\bar{r}$ could alternatively be calculated as the instantaneous average growth rate over all haplotypes in the population, as typically used in population genetics (Crow & Kimura, 1977). In this case, Equations 11, 14 and 16 would become respectively:

$$\begin{aligned} \bar{r}= r_{1}\theta_{1}+r_{2}\theta_{2}+\ldots+r_{M}\theta_{M}\#\left( S.11 \right) \end{aligned}$$

$$\begin{aligned} \bar{r_{y1}}=\sum_{i=1}^{M} {\theta_{i}}_{\mathrm{start}}\bar{r_{i}}=\frac{1}{T}\sum_{i=1}^{M} {\theta_{i}}_{\mathrm{start}}\ln\left( G_{i} \right)\#\left( S.12 \right) \end{aligned}$$

$$\begin{aligned} \bar{r_{y2}}=\sum_{i=1}^{M} {\theta_{i}}_{\mathrm{end}}\bar{r_{i}}=\frac{1}{T}\sum_{i=1}^{M} {\theta_{i}}_{\mathrm{end}}\ln\left( G_{i} \right)\#\left( S.13 \right) \end{aligned}$$

This approach gives minor differences in the calculated values of $\bar{r}$ and $\Delta\bar{r}$, but does not change the qualitative results presented. For example, compare Figure 5 (main paper, based on the mean growth rate over the cropping season), and Figure S.3 which shows the same qualitative results when $\bar{r}$ and $\Delta\bar{r}$ are calculated using the instantaneous growth rate as in Equation S.11.

S.1.4. Lande’s model of the effect of genetic variance on the rate of selection

Lande (1975, 1976, 1979) developed a model for the selection of a phenotypic character of individuals of a population that is under the control of a large number of genes. This model can also apply to a character, *Z*, that is the result of a large number of changes in one gene, as is the case for azole resistance. The model shows that the change in the numerical value of the character, Δ*Z*, over one generation is the product of the genetic variance, *G*, and the selection gradient, ∇*r*:

$$\begin{aligned} \Delta Z=G \nabla r\#\left( S.14 \right) \end{aligned}$$

For the case described here, *Z* is the average ED_50_ of the pathogen population to an azole fungicide, which changes over time due to selection through application of the azole. ∇*r*, the selection gradient, is the change in the average population growth rate caused by a change in the ED_50_ of the population.

Although Lande’s model can be applied to the evolution of azole resistance, it does not explicitly account for the effects of dose decay on differences in haplotype growth rates over time. The population genetic model that we have introduced here models dose decay explicitly, giving greater flexibility to model a wide range of azole programmes. However, in combination with Fisher’s (1930) classic text, Lande’s model provides a useful insight of the role of genetic variance in driving the rate of selection.

**References**

Crow J.F. & Kimura M. (1977). An Introduction to Population Genetics Theory. The Blackburn Press New Jersey. 591pp.

Fisher, R.A. (1930). The genetical theory of natural selection. Clarendon Press. https://doi.org/10.5962/bhl.title.27468

Hargrove, T.Y., Wawrzak, Z., Fisher, P.M., Child, S.A., Nes, W.D., Guengerich, F.P. et al. (2018). Binding of a physiological substrate causes large-scale conformational reorganization in cytochrome P450 51. *Journal of Biological Chemistry* 293, 19344–19353. https://doi.org/10.1074/jbc.RA118.005850

Lande, R. (1975). The maintenance of genetic variability by mutation in a polygenic character with linked loci. *Genetic Research* 26: 221-235.

Lande, R. (1976). Natural selection and random genetic drift in phenotypic evolution. *Evolution* 30: 314-334.

Lande, R. (1979). Quantitative genetic analysis of a multivariate evolution, applied to brain: body size allometry. *Evolution* 33: 402-416.

Parker, J.E., Warrilow, G.S., Cools, H.J., Martel, C.M., Nes, W.D., Fraajie, B.A. et al. (2011). Mechanism of binding of prothioconazole to *Mycosphaerella graminicola* CYP51 differs from that of other azole antifungals. *Applied and Environmental Microbiology* 77: 1460-1465. https://doi.org/10.1128/AEM.01332-10

Wang, N.S. (2014). Enzyme inhibition. Course notes.

https://terpconnect.umd.edu/~nsw/chbe482/inhibit.pdf [Accessed 17 September 2024].
