# Supplementary material for: Modelling Resistance Management Benefits of Incomplete Cross‐Resistance Within a Fungicide Mode of Action Group
Source: Evol Appl. 2026 Nov 5;19(11):e70346. doi: 10.1111/eva.70346 (PMC13736046; doi:10.1111/eva.70346)
Supplement: Supplementary file 2 [file EVA-19-e70346-s003.docx]

**Supplementary Material S2: Further details on model parameterisation**

S.2.1. Foliar dissipation half-life of azoles

There were no significant differences in the mean foliar dissipation half-lives (‘DT50’) of the azoles tebuconazole, epoxiconazole, prochloraz and prothioconazole-dethio as determined by a one-way ANOVA (F(3,15) = 0.06, p=0.979), based on DT50 estimates found in public domain sources (Table S.1).

Table S.1: Estimates of foliar dissipation half-life (‘DT50’) of tebuconazole, epoxiconazole, prochloraz and prothioconazole-desthio available in public domain sources.

| **Fungicide** | **Foliar half-life (days): values in published sources** | | | | | | **Mean** |
| --- | --- | --- | --- | --- | --- | --- | --- |
| Tebuconazole | 6.4^a^ | 7.7^b^ | 7.75^c^ | 7.76^d^ | 7.83^e^ | 10.12^f^ | 7.93 |
| Epoxiconazole | 3.1^g^ | 5.25^h^ | 5.3^i^ | 9.84^j^ | 13.31^d^ |  | 7.36 |
| Prochloraz | 3.4^k^ | 3.5^l^ | 6.85^m^ | 10.2^n^ | 11.5^o^ |  | 7.09 |
| Prothioconazole-desthio | 2.9^c^ | 6.7^p^ | 12.21^q^ |  |  |  | 7.27 |

Published sources indicated by letter superscripts. ^a^Dong & Hu, 2014; ^b^Lin et al., 2014; ^c^Lehoczki-Krjak et al., 2012; ^d^Fantke et al., 2014; ^e^Feng et al., 2020; ^f^He et al., 2010; ^g^Rao et al., 2013; ^h^Wu et al., 2017; ^i^Yan et al., 2015; ^j^Liu et al., 2007; ^k^Han et al., 2005. ^l^Liu et al., 2009; ^m^Feng et al., 2019; ^n^Yuan et al., 2016. ^o^Anonymous, 1983. ^p^Dong et al., 2019. ^q^Lin et al., 2017.

S.2.2. Initial haplotype frequencies

Figure S.3: Relative frequencies of CYP51 haplotype frequencies for which *in vitro* EC_50_ dose-response measurements were available, as sampled from the *Zymoseptoria tritici* population in fields around Rothamsted Research, Harpenden, UK, in 2010 to 2019. Haplotypes that reached a minimum frequency of 0.15 in one or more years from 2010 to 2019 shown.

S.2.3. Relationships between lab-EC_50_ and field-ED_50_ values

We used relationships between lab-EC_50_ and field-ED_50,_ developed by Blake et al. (2018), to convert lab-EC_50_ measurements of haplotype sensitivity (in (μg/ml)) to each azole into the field-ED_50_ values used for parameterisation of the $B_{ij}$ dose-response parameters for each haplotype-azole combination. For epoxiconazole y=0.3128x-0.007, where y=log(ED_50_) and x=log(EC_50_). For prothioconazole, y=3.812x+0.246 with y=log(prothioconazole ED_50_) and x=log(prothioconazole-desthio EC_50_). We used these relationships to calculate epoxiconazole and prothioconazole ED_50_ values respectively for each haplotype-azole combination. The fitted relationships differ between epoxiconazole and prothioconazole. We therefore assessed which, if any relationship was likely to be appropriate for converting tebuconazole and prochloraz EC_50_ values to ED_50_ values.

The antifungal action from an applied dose of prothioconazole is caused by its active metabolite prothioconazole-desthio (Parker et al., 2013). We compared the results of studies that measured the proportion of the applied dose of prothioconazole, epoxiconazole and tebuconazole that is successfully transferred onto or into plant leaves: the relevant comparison for prothioconazole is the proportion of applied prothioconazole that is transferred onto or into plant leaves as prothioconazole-desthio. When tebuconazole and prothioconazole were applied at the same rate, approximately 3.5g tebuconazole was measured on/in the flag leaf for every 1g of prothioconazole-desthio, a ratio of 3.5:1 (Lehoczki-Krsjak et al., 2013, 2015). From similar studies, we estimated this ratio as 4.7:1 for epoxiconazole vs. prothioconazole-desthio (Lichiheb et al., 2015; Lin et al., 2017). Applying this ratio as a correction factor explains the differences between epoxiconazole and prothioconazole in the observed relationships between EC_50_ and ED_50_. We therefore assumed that the relationship between EC_50_ and ED_50_ is consistent for other azole fungicides, and used the relationship between EC_50_ and ED_50_ for epoxiconazole as an estimate for the relationship between EC_50_ and ED_50_ for tebuconazole and prochloraz.

For any haplotypes which had a direct measurement of lab-EC_50_ for prothioconazole-desthio, we used the mean value of prothioconazole-desthio EC_50_ measurements across all isolates with that haplotype. However, seven haplotypes included in the analysis did not have EC_50_ measurements for prothioconazole-desthio, but did have EC_50_ measurements for prothioconazole (Table S.5, Supplementary Material S3). For some isolates EC_50_ measurements were available for both prothioconazole-desthio and prothioconazole (Table S.4, Supplementary Material S3). We fitted a relationship between the lab-EC_50_ measurements of prothioconazole-desthio and prothioconazole (Figure S.4) and used this to estimate prothioconazole-desthio EC_50_ from prothioconazole EC_50_ measurements in cases where there were no EC_50_ measurements for prothioconazole-desthio. For prothioconazole-desthio, y=0.7152x-4.3645, where y=log(prothioconazole-desthio EC_50_) and x=log(prothioconazole EC_50_).

Figure S.4: Fitted relationship between log(prothioconazole EC_50_) (log (μg/ml)) and log(prothioconazole-desthio EC_50_) (log (μg/ml)). Adjusted R^2^ = 63.95%.

S.2.4. Fitted dose response

The fitted dose response was compared to the published ‘protectant’ dose response curves for epoxiconazole and prothioconazole against *Z. tritici* shown in Blake et al. (2018). The model does not simulate disease on individual leaves as measured for ‘protectant situations’ in Blake et al. (2018), but we aimed to approximate a protectant application at the T1 timing (see Section 2.4). Model simulations were run using the initial haplotype starting frequency for each year (2003 and 2010-2019), for an untreated scenario and for each solo azole fungicide applied once at the T1 timing at a range of doses from 0.1 - 2 times the full label dose. The predicted severity at GS75 for each dose rate was averaged over four time periods (2003; 2010-2012; 2013-2015; 2016-2019), to correspond to those presented in Blake et al. (2018). The percentage disease control was then calculated from the predicted severity following fungicide application, compared to the scenario where the disease was left untreated. The mean predicted dose responses to epoxiconazole and prothioconazole over time correspond well overall to the mean ‘protectant’ dose responses shown in Blake et al. (2018) (Figure S.5).

Figure S.5: Mean fitted dose response over time for epoxiconazole, prochloraz, prothioconazole and tebuconazole.

**References**

Anonymous (1983). Data and recommendations of the joint meeting of the FAO Panel of Experts on Pesticide Residues in Food and the Environment and the WHO Expert Group on Pesticide Residues Geneva, 5 - 14 December 1983. Available at:

https://www.inchem.org/documents/jmpr/jmpmono/v83pr37.htm [Accessed 14 June 2025].

Blake, J.J., Gosling, P., Fraaije, B.A., Burnett, F.J., Knight, S.M., Kildea, S. et al. (2018). Changes in field dose–response curves for demethylation inhibitor (DMI) and quinone outside inhibitor (QoI) fungicides against *Zymoseptoria tritici*, related to laboratory sensitivity phenotyping and genotyping assays. *Pest Management Science* 74, 302–313.

https://doi.org/10.1002/ps.4725

Dong, B. & Hu, J. (2014). Dissipation and residue determination of fluopyram and tebuconazole residues in watermelon and soil by GC-MS. *International Journal of Environmental Analytical Chemistry*, *94*(5), 493–505.

https://doi.org/10.1080/03067319.2013.841152

Dong, X., Tong, Z., Chu, Y., Sun, M., Wang, M., Gao, T. et al. (2019). Dissipation of Prothioconazole and Its Metabolite Prothioconazole-Desthio in Rice Fields and Risk Assessment of Its Dietary Intake. *Journal of Agricultural and Food Chemistry*, *67*(23), 6458–6465. https://doi.org/10.1021/acs.jafc.8b06788

Fantke, P., Gillespie, B.W., Juraske, R. & Jolliet, O. (2014). Estimating Half-Lives for Pesticide Dissipation from Plants. *Environmental Science & Technology*, *48*(15), 8588–8602.

https://doi.org/10.1021/es500434p

Feng, Y., Han, J., Qi, X., Wang, X., Pan, J., Jin, J. et al. (2019). Residue and Safety Use of 45% Prochloraz EW in Ginger. *Plant Disease and Pests* 10(5/6):28-30. https://doi.org/10.19579/j.cnki.plant-d.p.2019.05-06.008

Feng, Y., Qi, X., Wang, X., Liang, L. & Zuo, B. (2020). Residue dissipation and dietary risk assessment of trifloxystrobin, trifloxystrobin acid, and tebuconazole in wheat under field conditions. *International Journal of Environmental Analytical Chemistry*, *102*(7), 1598–1612. https://doi.org/10.1080/03067319.2020.1739667

Han, L., Qian, C., Jiang, C. & Wang, W. (2005). Residue Detection and Degradation of Prochloraz and its Metabolites in Rice. *Chinese Journal of Pesticide Science* 7(1): 54-58.

He, L., Gong, D., Zuo, C. & Ma, H. (2010). Residue and degradation dynamics of tebuconazole SYP-1620 20%SC in rice field. *Agrochemicals* 7:506-509.

Lehoczki-Krsjak, S., Varga, M. & Mesterházy, Á. (2015). Distribution of prothioconazole and tebuconazole between wheat ears and flag leaves following fungicide spraying with different nozzle types at flowering. *Pest Management Science*, 71(1), 105–113.

https://doi.org/10.1002/ps.3774

Lehoczki-Krsjak, S., Varga, M., Szabó-Hevér, Á. & Mesterházy, Á. (2013). Translocation and degradation of tebuconazole and prothioconazole in wheat following fungicide treatment at flowering. *Pest Management Science*, 69(11), 1216–1224. https://doi.org/10.1002/ps.3486

Lichiheb, N., Bedos, C., Personne, E., Benoit, P., Bergheaud, V., Fanucci, O. et al. (2015). Measuring Leaf Penetration and Volatilization of Chlorothalonil and Epoxiconazole Applied on Wheat Leaves in a Laboratory-Scale Experiment. *Journal of Environmental Quality*, *44*(6), 1782–1790. https://doi.org/10.2134/jeq2015.03.0165

Lin, H., Dong, B. & Hu, J. (2017). Residue and intake risk assessment of prothioconazole and its metabolite prothioconazole-desthio in wheat field. *Environmental Monitoring and Assessment*, *189*(5), 236. https://doi.org/10.1007/s10661-017-5943-1

Lin, J., Zhang, Y., Han, B.-J. & Lyu, D. (2014). Residue dynamics of tebuconazole in different tissues of banana. *Journal of Southern Agriculture* 45(9), 1599. doi.10.3969/j:issn.2095-1191.2014.9.1599

Liu, X., Shen, M., Li, S. & Chen, H. (2009). Residue Dynamics of Prochloraz and its Metabolite2,4,6-Trichlorophenol in Mushroom and Soil. *Chinese Journal of Pesticide Science* 11(3): 362-366

Liu, L., Xu, Y., Huang, Y., Sun, Y. & Lin, D. (2007). Residue Dynamics of Epoxiconazole in Apples and Soil. *Modern Scientific Instruments*, 1: 61-63.

Parker, J. E., Warrilow, A. G., Cools, H. J., Fraaije, B. A., Lucas, J. A., Rigdova, K. et al. (2013). Prothioconazole and prothioconazole-desthio activities against *Candida albicans* sterol 14-α-demethylase. *Applied and environmental microbiology*, *79*(5), 1639–1645.

https://doi.org/10.1128/AEM.03246-12

Rao, T. N., Reddy, E. S., Reddy, G. R., Sreenivasulu, D. & Ramesh, J. (2013). Persistence study of pyraclostrobin and epoxiconazole fungicide formulation in groundnut plant followed by HPLC-UV method. *International journal of current microbiology and applied sciences*, *2*(9), 5-13.

Wu, X., Ma, J., Wang, H., Zhou, L., Li, T., An, L. et al. (2017). Residue and dissipation of epoxiconazole in *Triticum aestivum* L. and soil under field conditions. *Chinese Journal of Pesticide Science*, 2017, 19(4): 474-481.

https://doi.org/10.16801/j.issn.1008-7303.2017.0062

Yan, B., Ye, F. & Gao, D. (2015). Residues of the fungicide epoxiconazole in rice and paddy in the Chinese field ecosystem. *Pest Management Science*, *71*(1), 65–71.

https://doi.org/10.1002/ps.3763

Yuan, X.-Y., Peng, Z., Zhou, H.-P. & Liu, C. (2016). Degradation dynamics and safety evaluation of prochloraz in banana. *Journal of Food Safety and Quality* 7(1): 209-214.
