# Supplementary material for: Modelling Resistance Management Benefits of Incomplete Cross‐Resistance Within a Fungicide Mode of Action Group
Source: Evol Appl. 2026 Nov 5;19(11):e70346. doi: 10.1111/eva.70346 (PMC13736046; doi:10.1111/eva.70346)
Supplement: Supplementary file 3 [file EVA-19-e70346-s005.docx]

**Supplementary Material S5: Further details on model results**

S.5.1 Further details on the interpretation of $\Delta\bar{r}$ and its relationship with frequency-weighted ED_50_

The effect of a shift in the frequency-weighted ED_50_ of the entire pathogen population on disease control will vary depending on the initial level of disease control provided by the fungicide. Therefore a large shift in ED_50_ could result in a high or moderately high value of $\Delta\bar{r}$, depending on the impact of the shift on disease control. If the initial ED_50_ prior to selection is low, a large shift in ED_50_ will lead to a large change in the level of disease control, measured by a high value of $\Delta\bar{r}$. However, if the initial ED_50_ prior to selection is high (i.e. the fungicide already provides relatively low disease control even at the full label dose), a large shift in ED_50_ will only have a moderate impact on the epidemic growth rate, measured by a moderately high value of $\Delta\bar{r}$. Table S.7 compares the values of $\Delta\bar{r}$, the shift in frequency-weighted ED_50_ and the shift in disease control for a number of example cases, covering a range from low to high $\Delta\bar{r}$ values and small to large ED_50_ shifts.

Table S.7: Comparison of $\Delta\bar{r}$ and ED_50_ shift as measures of the development of resistance, for a number of example cases covering a range from low to high $\Delta\bar{r}$ values and small to large ED_50_ shifts. $\bar{r_{\mathrm{Start}}}$ and $\bar{r_{\mathrm{End}}}$ denote, respectively, the average growth rate of the entire pathogen population at the start of year 1 and at the end of year 2 of the fungicide programme. $\bar{{ED}_{{50}_{\mathrm{Start}}}}$ and $\bar{{ED}_{{50}_{\mathrm{End}}}}$ denote, respectively, the frequency-weighted ED_50_ of the entire pathogen population at the start of year 1 and at the end of year 2 of the fungicide programme. The % disease control is the predicted percentage disease control provided by a single application at full dose rate at T1, compared to an untreated epidemic.

| **Fungicide programme and year**  **(initial haplotype starting frequencies)** | $\boldsymbol{\Delta}\bar{\boldsymbol{r}}$ | $\bar{\boldsymbol{r}_{\mathbf{Start}}}$  **(**$\boldsymbol{t}^{\boldsymbol{-1}}$**)** | $\bar{\boldsymbol{r}_{\mathbf{End}}}$  **(**$\boldsymbol{t}^{\boldsymbol{-1}}$**)** | $\bar{\boldsymbol{ED}_{\boldsymbol{50}_{\mathbf{Start}}}}$ | $\bar{\boldsymbol{ED}_{\boldsymbol{50}_{\mathbf{End}}}}$ | $\bar{\boldsymbol{ED}_{\boldsymbol{50}}}$ **shift** | **% disease control, T1 application (Start)** | **% disease control, T1 application (End)** |
| --- | --- | --- | --- | --- | --- | --- | --- | --- |
| Prochloraz (solo), 2016 | 0.0060 | 0.0810 | 0.0814 | 0.604 | 0.617 | 0.013 | 65.5 | 64.9 |
| Epoxiconazole (solo), 2014 | 0.0075 | 0.0886 | 0.0893 | 0.872 | 0.903 | 0.031 | 56.2 | 55.3 |
| Prothioconazole (solo), 2013 | 0.0515 | 0.0828 | 0.0870 | 0.720 | 0.835 | 0.115 | 64.3 | 58.6 |
| Tebuconazole (solo), 2014 | 0.0532 | 0.0954 | 0.1005 | 1.370 | 1.856 | 0.486 | 47.6 | 38.2 |
| Prothioconazole (solo), 2016 | 0.1034 | 0.0883 | 0.0974 | 0.861 | 1.126 | 0.265 | 57.8 | 44.8 |
| Prochloraz (solo), 2010 | 0.1379 | 0.0743 | 0.0892 | 0.378 | 0.572 | 0.195 | 76.6 | 68.3 |
| Prothioconazole (solo), 2011 | 0.2016 | 0.0699 | 0.0796 | 0.540 | 0.845 | 0.305 | 73.2 | 58.1 |

S.5.2 Further details on case study of two-way mixtures of epoxiconazole and prochloraz

Table S.8 compares the effects of application of epoxiconazole (solo), prochloraz (solo) or a 50:50 mixture of epoxiconazole and prochloraz over two years on CYP51 haplotypes frequencies, fungicide programme efficacy and the rate of selection for resistance (as measured by $\Delta\bar{r}$.

Out of the three fungicide programmes, prochloraz (solo) gave the highest level of control, both against the initial haplotype composition and after two years of selection. However, erosion of the level of control was also fastest for prochloraz (solo). The level of control achieved with the mixture programme was slightly lower, but more stable due to the lower rate of selection for resistance. Epoxiconazole (solo) was not predicted to meet the threshold for effective disease control (Section 2.1.2).

The average programme efficacy of each programme against the initial haplotype composition and after two years of selection is measured by the values of $\bar{f}_{\mathrm{start}}$ and $\bar{f}_{\mathrm{end}}$. The level of control obtainable with prochloraz (solo) was predicted to be greater after two years’ use of the mixture programme compared to two years’ use of prochloraz (solo), suggesting that use of azole mixtures could help to protect the efficacy of the individual components of the mixture. In contrast, there would be little difference in the level of control obtainable with the mixture following two years’ use of mixture versus two years’ use of prochloraz (solo).

For this case study the proportion of each azole j in the mixture was simulated in 0.05 increments between 0.05 j1 + 0.95 j2 to 0.95 j1 + 0.05 j2. The proportion of each azole in the mixture affected both the rate of selection and the level of disease control (Figure S.6). The lowest rate of selection, measured by ∆r ̅, was achieved with mixtures between a 50:50 prochloraz to epoxiconazole ratio and a 25:75 prochloraz to epoxiconazole ratio; the 50:50 ratio provided better disease control than a 25:75 ratio. A 75:25 prochloraz to epoxiconazole ratio improved disease control, but at the cost of a higher rate of selection.

Figure S.6: Variation in the rate of resistance development, $\Delta\bar{r}$, and epidemic severity with the ratio of two azoles in a mixture: prochloraz and epoxiconazole, for a *Z. tritici* population with the CYP51 haplotype frequencies sampled in 2015 in Harpenden, UK. Points are plotted as pie charts indicating the ratio of prochloraz and epoxiconazole in the mixture: prochloraz – purple; epoxiconazole – orange. In addition to solo prochloraz and solo epoxiconazole, the proportion of each azole *j* in the mixture was simulated in 0.05 increments between 0.05 *j_1_* + 0.95 *j_2_* to 0.95 *j_1_* + 0.05 *j_2_*; points shown here are for 0.25 increments for visual clarity.

Table S.8: Comparison of the effects of two years of application of epoxiconazole (solo), prochloraz (solo) or a 50:50 mixture of epoxiconazole and prochloraz on selection of individual CYP51 haplotypes, and the overall resulting change in fungicide programme efficacy, for initial haplotype frequencies based on a sample from 2015 from Harpenden, UK. $\bar{f}_{\mathrm{start}}$ is a measure of average programme efficacy weighted by the initial haplotype frequencies: the average fractional reduction of the pathogen growth rate at the applied dose rate (Equation 20). $\Delta\bar{r}$ measures the rate of change of the pathogen population growth rate for each programme (Equation 1). $\bar{f}_{\mathrm{end}}$ is the average programme efficacy weighted by the final haplotype frequencies following two years of fungicide application.

| **Haplotype** | **Initial frequency (%)** | **ED_50_** | | **Fungicide programme** | | |
| --- | --- | --- | --- | --- | --- | --- |
|  |  |  |  | **Epoxiconazole** | **Prochloraz** | **Mixture** |
|  |  | **Epoxiconazole** | **Prochloraz** | **Percentage change in frequency** | | |
| L50S-V136A-Y461S | 3.16 | 0.579 | 0.799 | -66.5 | **+74.7** | -18.3 |
| L50S-D134G-V136G-Y461S | 3.16 | 0.782 | 0.642 | -33.2 | **+6.7** | -8.7 |
| L50S-D134G-V136A-I381V-Y461H | 33.68 | 0.823 | 0.580 | -25.5 | -16.2 | -15.9 |
| L50S-S188N-I381V-DEL-N513K | 36.84 | 0.974 | 0.634 | **+5.5** | **+3.7** | **+10.4** |
| L50S-S188N-A379G-I381V-DEL-N513K | 3.16 | 0.755 | 0.293 | -38.3 | -85.6 | -73.9 |
| L50S-V136A-S188N-DEL-S524T | 3.16 | 0.784 | 0.990 | -32.9 | **+171.9** | **+45.9** |
| L50S-V136A-I381V-Y461H-S524T | 5.26 | 1.122 | 0.686 | **+38.3** | **+24.4** | **+37.8** |
| L50S-D134G-V136A-I381V-Y461H-S524T | 5.26 | 1.281 | 0.675 | **+75.6** | **+19.7** | **+49.0** |
| L50S-V136C-S188N-I381V-Y461H-S524T | 3.16 | 1.170 | 0.494 | **+49.4** | -43.3 | -11.6 |
| L50S-V136C-S188N-A379G-I381V-DEL-S524T | 3.16 | 1.550 | 0.394 | **+140.2** | -68.2 | -29.9 |
| ${\bar{\boldsymbol{f}}}_{\mathbf{start}}$ **for fungicide programme** | | | | 0.466 | 0.570 | 0.526 |
| $\bar{\boldsymbol{r}}$ **for fungicide programme at start of year 1 (**$\boldsymbol{t}^{\boldsymbol{-}\boldsymbol{1}}$**)** | | | | 0.0900 | 0.0813 | 0.0850 |
| $\bar{\boldsymbol{r}}$ **for fungicide programme at end of year 2 (**$\boldsymbol{t}^{\boldsymbol{-}\boldsymbol{1}}$**)** | | | | 0.0913 | 0.0828 | 0.0856 |
| $\boldsymbol{\Delta}\bar{\boldsymbol{r}}$ | | | | 0.0148 | 0.0182 | 0.0071 |
| ${\bar{\boldsymbol{f}}}_{\mathbf{end}}$ **for epoxiconazole (solo)** | | | | 0.448 | 0.470 | 0.461 |
| ${\bar{\boldsymbol{f}}}_{\mathbf{end}}$ **for prochloraz (solo)** | | | | 0.573 | 0.553 | 0.562 |
| ${\bar{\boldsymbol{f}}}_{\mathbf{end}}$ **for mixture** | | | | 0.522 | 0.517 | 0.518 |

S.5.3 Variation in $\Delta\bar{r}$ of mixture programmes over time and impact of individual azole active substances

Table S.9: Azole programmes meeting the threshold for effective control that tended to minimise or maximise $\Delta\bar{r}$ most frequently for the scenarios (i.e. sampled haplotype starting frequencies by year) modelled, and range of values of cross-resistance (*cc_w_),* programme efficacy ($\bar{f}_{start}$) and maximum variance in haplotype sensitivity ($\mathrm{Var}_{j_{\mathrm{Max}}}$) over the years relevant for each programme. ^1^Programmes were defined as maximising or minimising $\Delta\bar{r}$ in a year if they had one of the highest 3 or lowest 3 $\Delta\bar{r}$ values respectively within the year of programmes meeting the threshold for effective control. In years when fewer than 6 programmes met threshold for effective control (2016, 2018, 2019), programmes with the highest/lowest 1 or 2 $\Delta\bar{r}$ values were considered to maximise/minimise $\Delta\bar{r}$ respectively. ^2^Prochloraz minimised $\Delta\bar{r}$ in 2016.

| **Fungicide programme** | **^1^Years minimised / maximised** $\boldsymbol{\Delta}\bar{\boldsymbol{r}}$ | | | ***cc_w_*** | ${\bar{\boldsymbol{f}}}_{\mathbf{start}}$ | $\mathbf{Var}_{\boldsymbol{j}_{\mathbf{Max}}}$  **(**$\boldsymbol{t}^{\boldsymbol{-}\boldsymbol{2}}$**)** |
| --- | --- | --- | --- | --- | --- | --- |
| **Programmes that tended to minimise** $\boldsymbol{\Delta}\bar{\boldsymbol{r}}$ | |  | | |  |  |
| Prochloraz, Epoxiconazole & Tebuconazole | 2003, 2010-2014 | | | -0.241 - 0.169 | 0.514 - 0.578 | 0.0087 - 0.0167 |
| Prochloraz & Epoxiconazole | 2003, 2014-2019 | | | -0.601 - 0.309 | 0.513 - 0.598 | 0.0021 - 0.0070 |
| Four-way mixture | 2012-2015 | | | 0.114 - 0.204 | 0.507 - 0.564 | 0.0101 - 0.0274 |
| **Programmes that tended to maximise** $\boldsymbol{\Delta}\bar{\boldsymbol{r}}$ | | |  | |  |  |
| Prothioconazole (solo) | 2003, 2010-2015, 2017 | | | 1 | 0.516 - 0.692 | 0.0036 - 0.0274 |
| Prothioconazole & Prochloraz | 2010-2019 | | | -0.061 - 0.656 | 0.522 - 0.687 | 0.0101 - 0.0274 |
| ^2^Prochloraz (solo) | 2010-2014, 2017, 2019 | | | 1 | 0.590 - 0.689 | 0.0038 - 0.0107 |

Figure S.7: Variation in model results for solo and two-way mixture azole programmes over time. (a). $\Delta\bar{r}$ for solo azole programmes over time. (b). $\Delta\bar{r}$ for two-way azole mixture programmes over time. (c). Predicted disease severity (%) at GS75 for solo and two-way mixture programmes over time. (d). Cross-resistance of azole pairs over time.

Figure S.8: Variation in the rate of resistance development, $\Delta\bar{r}$, with the number of azoles included in the mixture (‘within-MoA diversity’), excluding prochloraz from the analysis (for comparison with the results presented in Figure 4). (a) ). $\Delta\bar{r}$ over 2 years of application and level of disease control, measured as severity (%) at GS75 in the first year of application for all azole mixture programmes and initial haplotype frequency scenarios (years) modelled. The target for resistance management is programmes that combine effective disease control and a low rate of resistance development, $\Delta\bar{r}$. Number of azoles in each programme indicated by: 1 azole: black circle; 2 azoles: purple cross; 3 azoles: blue triangle. The 5% severity threshold for effective disease control is indicated by the grey dotted line. (b) Variation with number of azoles and time of the mean $\Delta\bar{r}$ of programmes meeting the threshold for effective disease control (<5% severity at GS75). The mean $\Delta\bar{r}$ of groups of years is indicated as follows: 2003: orange solid line; 2010-2012: black dashed line; 2013-2015: purple dotted line. Note that out of the fungicide programmes modelled, only programmes containing prochloraz met the threshold for effective disease control in 2016-2019, so there is no data to show for this time period when prochloraz is excluded from the analysis. Error bars show ±1 standard error of the mean; in cases where only one azole programme in the category met the threshold for effective disease control, these are plotted as points without an error bar. Averages are joined by point-to-point lines to aid interpretation.

S.5.4 Further details on generalised linear models of $\Delta\bar{r}$

We fitted separate GLMs for each measure of cross-resistance to determine which was most informative (Table S.10). The measure of cross-resistance was the first explanatory variable term included in each model, with the order of other terms determined through forward stepwise regression.

Table S.10: Percentage variance in $\Delta\bar{r}$ explained by individual variates included in generalised linear models (GLMs) with a log link function for 1, 2, 3 and 4-azole programmes. Explanatory variables are listed in the order that terms were added to each GLM: cross-resistance was always included first, whilst the inclusion and order of other terms was determined by forwards stepwise regression.

| **Number of azoles in programme** | **Explanatory variable** | **% variance explained by variate in GLM** | | |
| --- | --- | --- | --- | --- |
|  |  | **^1^Measure of cross-resistance** | | |
|  |  | ***cc_w_*** | ***cc_u_*** | ***cc_z_*** |
| 1 | Cross-resistance | ^4^N/A | ^4^N/A | ^4^N/A |
|  | ^3^$\mathrm{Var}_{j_{\mathrm{Max}}}$ | 44.6 |  |  |
|  | ^2^$\bar{f}_{\mathrm{start}}$ | 36.4 |  |  |
| 2 | Cross-resistance | 36.6 | 22.9 | 11.3 |
|  | ^2^$\bar{f}_{\mathrm{start}}$ | 35.0 | 38.5 | 46.6 |
|  | ^3^$\mathrm{Var}_{j_{\mathrm{Max}}}$ | 12.1 | 14.0 | 13.5 |
| 3 | Cross-resistance | 38.6 | 25.6 | 7.8 |
|  | $\bar{f}_{\mathrm{start}}$ | 40.2 | 46.2 | 61.9 |
|  | $\mathrm{Var}_{j_{\mathrm{Max}}}$ | 6.4 | 6.6 | 5.7 |
| 4 | Cross-resistance | 36.0 | 42.4 | 1.6 (^5^NS) |
|  | $\bar{f}_{\mathrm{start}}$ | 48.7 | 42.1 | 85.5 |
|  | $\mathrm{Var}_{j_{\mathrm{Max}}}$ | 7.1 | 0.0 (^5^NS) | 0.6 (^5^NS) |

^1^Measures of cross-resistance calculated using ED_50_ field doses are the frequency-weighted correlation coefficient, unweighted Pearson’s correlation coefficient and the Spearman’s rank correlation coefficient, denoted as *cc_w_*, *cc_u_* and *cc_z_* respectively (see Section 2.5.1).
^2^Average programme efficacy measured as $\bar{f}_{\mathrm{start}}$, the average fractional reduction of the pathogen growth rate at the applied dose rate, weighted by the initial haplotype frequencies (see Section 2.5.2).

^3^Maximum variance in haplotype sensitivity for a single azole component of the mixture, measured as $\mathrm{Var}_{j_{\mathrm{Max}}}$(see Section 2.5.3).

^4^Cross-resistance is not an informative term for 1-azole programmes, as the cross-resistance of an azole with itself is always equal to 1.

^5^NS denotes terms that were not significant (F-test). All other terms shown were significant.

We additionally fitted individual GLMs for each explanatory variable (Table S.11) and checked the level of correlation between explanatory variables. We checked for significant interactions between each of the explanatory variables and the number of azoles included in each programme, to determine whether separate GLMs should be fitted for solo azoles, two-way, three-way and four-way mixtures. There were significant interactions between the number of azoles and the fitted generalised linear model (GLM) coefficients for $\bar{f}_{\mathrm{start}}$ and $\mathrm{Var}_{j_{\mathrm{Max}}}$ (Tables S.12, S.13). The percentage of the variance in $\Delta\bar{r}$ explained by $\bar{f}_{\mathrm{start}}$ as an individual variable increased with the number of azoles included in the mixture (Table S.11). For 2-azole programmes, there was no significant correlation between the explanatory variables, but for 4-azole programmes, $\bar{f}_{\mathrm{start}}$ was positively correlated with ${cc}_{w}$ (Spearman’s correlation, *r_s_* (9) = 0.63, p=0.039).

The accumulated analysis of variance tables for GLMs of $\Delta\bar{r}$ for solo azoles, two-way, three-way and four-way mixtures are shown in Tables S.14, S.15, S.16 and S.17 respectively, and a comparison of the GLM fitted values and simulation model values of $\Delta\bar{r}$ for solo azoles is shown in Figure S.9.

Table S.11: Percentage variance in $\Delta\bar{r}$ of 1, 2, 3 and 4-azole programmes explained by individual variables in generalised linear models (GLMs) with a log-link function, with only one explanatory variable per GLM. ^1^Cross-resistance is not an informative term for 1-azole programmes, as the cross-resistance of an azole with itself is always equal to 1. ^2^NS denotes variables for which there was no significant correlation (F-test). All other variables shown were significant.

| **Number of azoles in programme** | **Explanatory variable** | **% variance explained as single variate** |
| --- | --- | --- |
|  |  |  |
|  |  |  |
| 1 | *cc_w_* | ^1^N/A |
|  | ^3^$\mathrm{Var}_{j_{\mathrm{Max}}}$ | 43.3 |
|  | ^2^$\bar{f}_{\mathrm{start}}$ | 24.8 |
| 2 | *cc_w_* | 35.7 |
|  | ^2^$\bar{f}_{\mathrm{start}}$ | 48.2 |
|  | ^3^$\mathrm{Var}_{j_{\mathrm{Max}}}$ | 7.6 |
| 3 | *cc_w_* | 37.1 |
|  | $\bar{f}_{\mathrm{start}}$ | 65.1 |
|  | $\mathrm{Var}_{j_{\mathrm{Max}}}$ | 0.0 (^2^NS) |
| 4 | *cc_w_* | 28.9 (^2^NS: p=0.051) |
|  | $\bar{f}_{\mathrm{start}}$ | 80.1 |
|  | $\mathrm{Var}_{j_{\mathrm{Max}}}$ | 0.0 (^2^NS) |

Table S.12: Accumulated analysis of variance for a generalised linear model of $\Delta\bar{r}$ with a log link function for two and three-azole mixture programmes.

| **Variable** | **d.f.** | **s.s.** | **m.s.** | **v.r.** | **F pr.** |
| --- | --- | --- | --- | --- | --- |
| *cc_w_* | 1 | 0.01483 | 0.01483 | 253.08 | <.001 |
| $\bar{f}_{\mathrm{start}}$ | 1 | 0.01367 | 0.01367 | 233.26 | <.001 |
| $\mathrm{Var}_{j_{\mathrm{Max}}}$ | 1 | 0.00346 | 0.00346 | 59.04 | <.001 |
| 2 vs. 3 azoles | 1 | 0.00092 | 0.00092 | 15.64 | <.001 |
| 3 azoles * $\bar{f}_{\mathrm{start}}$ | 1 | 0.00047 | 0.00047 | 7.96 | 0.006 |
| Residual | 104 | 0.00609 | 0.00006 |  |  |
| Total | 109 | 0.03943 | 0.00036 |  |  |

Table S.13: Accumulated analysis of variance for a generalised linear model of $\Delta\bar{r}$ with a log link function for 2-azole mixture programmes and solo azole programmes.

| **Variable** | **d.f.** | **s.s.** | **m.s.** | **v.r.** | **F pr.** |
| --- | --- | --- | --- | --- | --- |
| *cc_w_* | 1 | 0.01983 | 0.01983 | 99.82 | <.001 |
| $\bar{f}_{\mathrm{start}}$ | 1 | 0.03340 | 0.03340 | 168.15 | <.001 |
| $\mathrm{Var}_{j_{\mathrm{Max}}}$ | 1 | 0.04274 | 0.04274 | 215.16 | <.001 |
| 2 azoles vs. solo | 1 | 0.00219 | 0.00219 | 11.00 | 0.001 |
| Solo * $\mathrm{Var}_{j_{\mathrm{Max}}}$ | 1 | 0.00460 | 0.00460 | 23.17 | <.001 |
| Residual | 104 | 0.02066 | 0.00020 |  |  |
| Total | 109 | 0.12343 | 0.00113 |  |  |

Table S.14: Accumulated analysis of variance for a generalised linear model of $\Delta\bar{r}$ with a log link function for solo azole programmes.

| **Variable** | **d.f.** | **s.s.** | **m.s.** | **v.r.** | **F pr.** |
| --- | --- | --- | --- | --- | --- |
| $\mathrm{Var}_{j_{\mathrm{Max}}}$ | 1 | 0.03771 | 0.03771 | 96.24 | <.001 |
| $\bar{f}_{\mathrm{start}}$ | 1 | 0.03083 | 0.03083 | 78.67 | <.001 |
| Residual | 41 | 0.01607 | 0.00039 |  |  |
| Total | 43 | 0.08460 | 0.00197 |  |  |

Table S.15: Accumulated analysis of variance for a generalised linear model of $\Delta\bar{r}$ with a log link function for two-azole mixture programmes.

| **Variable** | **d.f.** | **s.s.** | **m.s.** | **v.r.** | **F pr.** |
| --- | --- | --- | --- | --- | --- |
| *cc_w_* | 1 | 0.01025 | 0.01025 | 139.96 | <.001 |
| $\bar{f}_{\mathrm{start}}$ | 1 | 0.00980 | 0.00980 | 133.74 | <.001 |
| $\mathrm{Var}_{j_{\mathrm{Max}}}$ | 1 | 0.00338 | 0.00338 | 46.19 | <.001 |
| Residual | 62 | 0.00454 | 0.00007 |  |  |
| Total | 65 | 0.02797 | 0.00043 |  |  |

Table S.16: Accumulated analysis of variance for a generalised linear model of $\Delta\bar{r}$ with a log link function for three-azole mixture programmes.

| **Variable** | **d.f.** | **s.s.** | **m.s.** | **v.r.** | **F pr.** |
| --- | --- | --- | --- | --- | --- |
| *cc_w_* | 1 | 0.00393 | 0.00393 | 103.77 | <.001 |
| $\bar{f}_{\mathrm{start}}$ | 1 | 0.00409 | 0.00409 | 108.09 | <.001 |
| $\mathrm{Var}_{j_{\mathrm{Max}}}$ | 1 | 0.00065 | 0.00065 | 17.24 | <.001 |
| Residual | 40 | 0.00152 | 0.00004 |  |  |
| Total | 43 | 0.01019 | 0.00024 |  |  |

Table S.17: Accumulated analysis of variance for a generalised linear model of $\Delta\bar{r}$ with a log link function for four-azole mixture programmes.

| **Variable** | **d.f.** | **s.s.** | **m.s.** | **v.r.** | **F pr.** |
| --- | --- | --- | --- | --- | --- |
| *cc_w_* | 1 | 0.00050 | 0.00050 | 30.59 | <.001 |
| $\bar{f}_{\mathrm{start}}$ | 1 | 0.00067 | 0.00067 | 41.43 | 0.006 |
| $\mathrm{Var}_{j_{\mathrm{Max}}}$ | 1 | 0.00010 | 0.00010 | 6.00 | 0.044 |
| Residual | 7 | 0.00011 | 0.00002 |  |  |
| Total | 10 | 0.00138 | 0.00014 |  |  |

Figure S.9: Comparison of epidemiological model values vs. GLM fitted values of $\Delta\bar{r}$ for solo azole applications (denoted as black circle); solid black line denotes 1:1 line.

The cross-resistance of an azole with itself is always equal to 1. Using a value of *cc_w_*=1, the fitted parameter values for 2-azole mixtures (Table 3, main paper) were a moderately good predictor for the rank order of the $\Delta\bar{r}$ values of solo azole programmes (Spearman’s correlation, *r_s_* (42) = 0.57, p<.001), but there was a tendency to underpredict the highest $\Delta\bar{r}$ values. The GLM fitted for solo azole programmes was a better predictor of the rank order of the $\Delta\bar{r}$ values of solo azole programmes (Spearman’s correlation, *r_s_* (42) = 0.71, p<.001).
